# Practical Three-Minute Synthesis of Acid-Coated Fluorescent Carbon Dots with Tuneable Core Structure

**DOI:** 10.1038/s41598-018-29674-2

**Published:** 2018-08-15

**Authors:** Stephen A. Hill, David Benito-Alifonso, Sean A. Davis, David J. Morgan, Monica Berry, M. Carmen Galan

**Affiliations:** 1 School of Chemistry, University of Bristol, Cantock’s Close, Bristol, BS8 1TS UK; 2 Cardiff Catalysis Institute, School of Chemistry, Cardiff University, Park Place, Cardiff, CF10 3AT UK; 3 School of Physics, University of Bristol, Bristol, BS8 1TL UK

## Abstract

We report a one-pot, three-minute synthesis of carboxylic acid-decorated fluorescent carbon dots (COOH-FCDs) with tuneable core morphology dependent on the surface passivating agent. Mechanism investigations highlighted the presence of key pyrazine and polyhydroxyl aromatic motifs, which are formed from the degradation of glucosamine in the presence of a bifunctional linker bearing acid and amine groups. The novel COOH-FCDs are selective Fe^3+^ and hemin sensors. Furthermore, the FCDs are shown to be non-toxic, fluorescent bioimaging agents for cancer cells.

## Introduction

In the last decade, nanotechnology has had a considerable impact on many research applications^1–3^. An important focus area has been the development of fluorescent, non-toxic nanomaterials that can be tracked *in vivo* and *in vitro*^4–8^, where fluorescent carbon dots (FCDs) provide an alternative to heavy metal-containing nanomaterials (e.g. quantum dots), due to their chemical inertness, high water solubility and very low cytotoxicity^9–16^. These photoluminescent carbon-based materials have found applications in for example gene delivery^9,17–19^, cell imaging^12^, metal sensing^11,20–22^, photo-catalysis^23^ and photovoltaics^23–26^. Due to their complex molecular structure, there is still a lack of fundamental understanding at the molecular level of their photoluminescence (PL) and chemical formation mechanisms, which has hampered the design of materials for specific applications^27^. Understanding the formation mechanism and interdependence between core and surface features has been the focus of this study.

**Figure 1:**
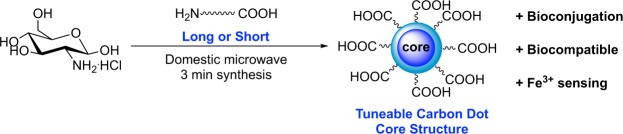
One-pot synthesis of carboxylic acid-coated carbon dots.

Although some microwave-assisted FCD syntheses are reported for a range of starting materials^28–32^.Most reported FCDs syntheses require relatively harsh conditions (chemical or hydrothermal oxidation, pyrolysis, high acidic environments and long reactions times), elaborate post-modifications to improve the surface state of the nanomaterials and lengthy purifications^12,33–35^, which makes the protocols difficult to handle by non-chemists at the point of use. Stemming from our interest in the development of facile protocols for the synthesis of fluorescent probes for biological applications^36–40^, we turned our attention to the preparation of water soluble FCDs from commercial starting materials that are ready for conjugation by a given biomolecule. Herein, we report the practical one-pot three-minute synthesis of carboxylic acid-functionalised FCDs (COOH-FCD) from a carbohydrate starting material and an amino acid (Fig. 1). Following full physical and chemical characterisation, we show that the FCD core structure is highly dependent on the surface passivating agent chosen, reflecting events in the formation of these FCDs. Moreover, low toxicity exhibited by the novel nanomaterials highlights potential applications in environmental sensing and as cellular imaging probes.

## Methods

### Materials and Characterisation Methods

Chemicals were purchased and used without further purification. COOH-FCD formation was conducted in a domestic microwave oven (Tesco Microwave Oven MM07, Input Power: 1200 W, Microwave Output: 700 W). Concentration centrifugation tubes were GE Healthcare Life Sciences VIVASPIN 6/20 with a 10,000 Da molecular-weight cut off filter. Extracts were concentrated under reduced pressure using both a Büchi rotary evaporator at a pressure of either 15 mmHg (diaphragm pump) and 0.1 mmHg (oil pump), as appropriate, and a high vacuum line at room temperature. ^1^H & ^13^C (HSQC and HMBC) NMR spectra were measured in D_2_O on 500 MHz Varian. ^1^H & ^13^C NMR chemical shifts are quoted in parts per million (ppm) and referenced to the residual solvent peak (D_2_O: ^1^H = 4.79 ppm); and coupling constants (J) given in Hertz. Multiplicities are abbreviated as: br (broad), s (singlet), d (doublet), t (triplet), q (quartet), p (pentet) and m (multiplet) or combinations thereof. Assignments were made, where necessary, with the aid of HSQC and HMBC NMR experiments. Kochetkov’s amination of unprotected carbohydrates was conducted using Biotage Initiator +microwave reactor. Fourier-transformed infra-red (FTIR) spectroscopy was conducted on a Bruker ATR. Dynamic Light Scattering (DLS) and Zeta analysis were carried out using Malvern Instruments, Nano-S90 Red Laser Model ZEN1690 for DLS and Nano-Z ZEN 2600 for Zeta potential. Zeta potential measurements were conducted in distilled H_2_O. The structure of CDs were was examined by transmission electron microscopy (TEM) on a Jeol 2100F with an accelerating voltage of 200 kV. A drop of the COOH-FCD methanol/water (1:2) suspension (5 mg/mL) was carefully applied to a 200-mesh carbon-coated copper grid and dried at ambient temperature for TEM characterization. TEM images were recorded using a Gatan SC1000 CCD camera and Gatan Microscopy Suite 3 (GMS 3) software. High resolution images were analyzed with GMS 3 to determine lattice spacings and ImageJ for particle sizing. Fluorescence measurements were made and conducted on a Perkin-Elmer LS45 in either 700 or 3500 µL quartz cuvettes (ThorLabs). Absorbance measurements were conducted on Cary UV-Vis 50 spectrophotometer in either 700 or 3500 µL quartz cuvettes (ThorLabs). Quantum yield of fluorescence (QY) measurements were conducted on both Perkin-Elmer LS45 and Cary UV-Vis 50 spectrophotometer in either 700 or 3500 µL quartz cuvettes (ThorLabs), relative to quinine sulfate (in 0.1 M H_2_SO_4_). X-ray photoelectron spectroscopy (XPS) was performed using a Kratos Axis Ultra DLD spectrometer, using monochromatic Al ka radiation operating at 144 W power (12 mA x 12 kV). For analysis, samples (dissolved in methanol/water (1:2)) were pipetted on to clean gold wafers and the solvent evaporated under vacuum in the fast-entry lock of the spectrometer. Analysis of all regions was taken at a pass energy of 20 eV with a 0.1 eV step for high resolution scans, and 150 eV and a 1 eV step for survey spectra. All data was subsequent calibrated to the lowest C(1s) line at 285 eV and quantified using sensitivity factors supplied by the manufacturer in CasaXPS v2.3.17PR1.1.

### Synthetic Procedures

#### Synthesis of 1-amino-15-oxo-4,7,10-trioxa-14-azaoctadecan-18-oic acid (TTA, 2)

To a solution of 4,7,10-trioxa-1,13-tridecanediamine (TTDDA) (1.00 g, 4.54 mmol) in MeCN (50 mL) was added portion-wise succinic anhydride (0.454 g, 4.54 mmol) with a high rate of stirring. The solution was left stirring for 17 h at room temperature. An oil-like precipitate was formed found underneath the bulk solvent. The residual MeCN was decanted and the resultant residue was reduced *in vacuo* to afford the mono acid 2 (1.39 g, 4.43 mmol) in a 95% yield. ^1^H Presat NMR (500 MHz, D_2_O) δ: 3.61–3.45 (10 H, d, e), 3.43 (2 H, t, J = 10 Hz, c), 3.12 (2 H, t, J = 10 Hz, g), 2.98 (2 H, t, J = 10 Hz, a), 2.50 (2 H, t, J = 10 Hz, j), 2.38 (2 H, t, J = 10 Hz, h), 1.79 (2 H, p, J = 5 Hz, b), 1.65 (2 H, p, J = 5 Hz, j); ^13^C NMR (125 MHz, D_2_O) δ: 26, 28, 32, 33, 36, 37, 68, 69, 70, 71, 174, 175 ppm MS-ESI for C_14_H_28_N_2_O_6_ calc: 320.19; found: 343.20 [M + Na^+^].

#### Synthesis of COOH-FCD (4 and 5)

To a stirring solution of glucosamine hydrochloride (1.00 g, 4.63 mmol) in distilled H_2_O (20 mL) in a 250 mL conical flask, 1-amino-15-oxo-4,7,10-trioxa-14-azaoctadecan-18-oic acid 2 (1.64 g, 5.09 mmol, for **4**) or β-alanine 3 (0.45 g, 5.09 mmol, for **5**) was added and stirred to ensure homogeneity. The conical flask (without the stirring bar) was then placed in a domestic microwave (in a fume cupboard) and the solution heated for 3 mins at 700 Watts (full power). A viscous brown residue was afforded, which was dissolved in distilled H_2_O (10 mL) and centrifuge-filtered through a GE Healthcare Life Sciences Vivaspin 20 MWCO 10000 spin column (8500 rpm, 30 mins). The filtrate was then reduced in vacuo (or lyophilised) to yield a thick brown oil, COOH-FCDs 4 or 5 (1–1.5 g).

#### Microwave-assisted Kochtekov’s Amination of Lactose

The microwave vial was charged with unprotected lactose (0.25 g), ammonium carbonate (5-fold excess w/w over lactose, 1.25 g) and anhydrous DMSO (0.8 mL). The mixture was purged with N_2_ and the tube was sealed and placed in an automated microwave at 600 C, 250 psi pressure and 10 watts power for 90 minutes. The reaction mixture was freeze dried overnight to remove excess ammonium carbonate and DMSO to afford β-glycosyl amines as hygroscopic solids. (1-aminolactose is typically a white or orange solid post-freeze drying). Conversion to aminoglycoside confirmed by Ninhydrin treatment.

#### Glyco-Functionalisation of COOH-FCD (6 and 7)

To COOH-FCDs 4 or 5 (10 mg) in distilled H_2_O (1 mL) was added CDI (1,1-carbonyldiimidazole, 40 mg) and the solution was sonicated for 15 min. Glycosylamine (lactose) 2 mL (10 mgmL^−1^ solution) was added to the solution and this was stirred for 17 h in the dark. The reaction mixture was dialysed against distilled H_2_O (500 MWCO) for 24 h (H_2_O changed regularly). The resulting solution was reduced *in vacuo* to yield a brown product, 6 or 7 (10–15 mg).

### Cell cultures, toxicity assays and confocal microscopy

#### Cell culture

MDA-MB-231 human breast cancer cells were grown in DMEM with 4.5 g glucose/L, GlutaMAX™ and 10% fetal bovine serum (FBS), and antibiotic-antimycotic solution (AntiAnti). Confluent cultures were dissociated using trypsin (Tryp LE Express) and plated at 10^4^ cells/well in either petri dishes (Mat-Tek 35 mm, with 14 mm glass microwell) for imaging, or 96-well plates for toxicity tests. All cell culture media and additives were purchased from Invitrogen, Life Technologies (Thermo-Fisher).

#### Toxicity assays

All tests were conducted with 8 replicates per condition, i.e. toxicant concentration and time point, after exposures of 1 h, 1 day, and 3 days. Experiments were repeated at least twice. FCDs were applied at final concentrations from 2000 to 10^−3^ µg mL^−1^ in medium with reduced FBS (5%), to allow cell division but avoid protection from high serum. The effects of **4** and **5** COOH-FCD on cell metabolism and viability were assessed using AlamarBlue and Calcein AM. AlamarBlue is cytosolic substrate (resazurin to resorufin) whose fluorescence spectrum changes on reduction. The number of live cells was evaluated using Calcein AM, which is enzymatically transformed into the fluorescent product Calcein in the cytoplasm of live cells. After exposure, cultures were washed with PBS, and incubated with both dyes simultaneously for 1 h. Fluorescence was measured on a CLARIOStar Microplate Reader at room temperature. Data are presented compared to control, unexposed cultures, on the same plate. Statistical analysis showed there was a significant interaction, between the concentration of either COOH-FCDs and the duration of exposure (p < 0.0001, Two Way ANOVA). The reductive metabolism per cell was significantly different for exposures of 1 h and 1 day, and 1 h and 3 days for either COOH-FCD (p < 0.001, corrected for multiple comparisons, respectively).

#### Confocal Microscopy

All images were acquired on a Leica SP5 confocal system equipped with a Leica DMI 6000 inverted microscope. For the excitation of the FCDs 405 nm excitation was used. Cells were live and imaged in Living Cell Imaging Solution (ThermoFisher Scientific). The images were analysed using Volocity software (PerkinElmer).

## Results and Discussions

### Synthesis of Acid functionalised FCDs

**Figure 2:**
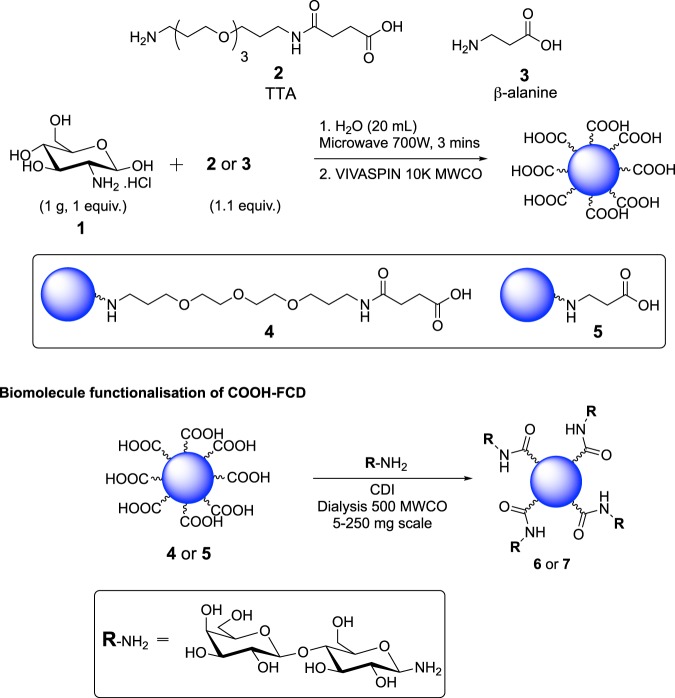
General synthetic preparation and lactose functionalization for COOH-FCDs **4** and **5**.

GlcNH_2_.HCl **1** was chosen as the C source as it already contains a N required for heteroatom doping. This is an important and widely used strategy for improving the QY of CDs, since the introduction of N-doping allows the injection of electrons into the CD structure, which allows for new PL and fluorescence properties to be established^27^. We hypothesised that the incorporation of a linker containing both a carboxylic acid and amine functionalities for surface passivation, would yield FCDs displaying surface acid groups, while maintaining PL properties. To that end, two different linkers bearing an amine and an acid group at each end, which differ in length and atomic nature, were chosen: 1-amino-15-oxo-4,7,10-trioxa-14-azaoctadecan-18-oic acid^41^ (TTA, long linker) **2**, which was prepared on a gram-scale after reaction of TTDDA with succinic anhydride in MeCN in 95% yield and commercial *β*-alanine **3** (short-linker). Successful gram-scale, microwave-assisted (domestic 700 W MW) synthesis of COOH-FCDs was carried out using GlcNH_2_.HCl **1** (1 equiv.) and either linker **2** or **3** (1.1 equiv.) in distilled water for 3 min. The reaction vessel is position always on the same spot on the MW plate to ensure batch-to batch reproducibility. Following dissolution in water and centrifugal filtration (10 kDa molecular weight cut-off) blue-fluorescent COOH-FCDs **4** and **5** were obtained (Figs 2 and S1 in ESI). To demonstrate the presence and accessibility of the surface-confined carboxylic acid groups, amide conjugation of **4** and **5** with 1-aminolactose using 1,1-carbonyldiimidazole (CDI) was performed and lactose-functionalised FCDs **6** and **7** were thus synthesised, as confirmed by ^1^H-NMR after dialysis purification (Fig. 2 and Figs S25 and S26 in ESI).

### Physical and Photoluminescence Characterisation

**Figure 3:**
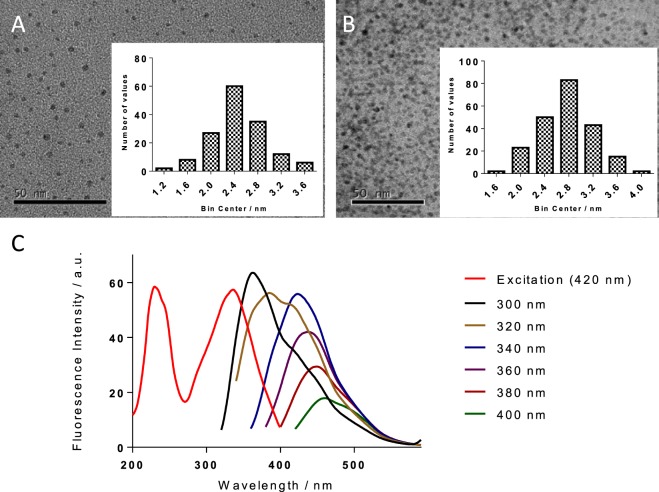
TEM images and sizing of (**A**) FCD **5** and (**B**) FCD **4** (number of values = number of particles); (**C**) excitation and emission spectrum of **4**.

High-resolution transmission electron microscopy (HR-TEM) showed differences between the two COOH-FCDs systems, with TTA-decorated **4** having an average diameter (and standard deviation) of 2.78 nm ± 0.42 (Fig. 3A,B) and 2.46 nm ± 0.44 for *β*-alanine-decorated **5**. The increase in core diameter in COOH-FCD **4** is attributed to increased linker incorporation within the nanoparticle. Dynamic light scattering (DLS) indicated a hydrodynamic diameter of approximately 2–6 nm for COOH-FCDs **4** and 5–10 nm for **5** which we attributed to differences in solvation of the two nanoparticles (Fig. S2A,B in ESI). Both samples exhibited lattice fringes (Fig. S3A,B in ESI) which could be assigned to a graphitic structure^42,43^ and is in agreement with previously reported carbohydrate-derived FCDs^43,44^. In both samples, spacings of 0.21 nm were most frequently observed corresponding to the (100) planes. Additional lattice fringes with interplanar spacings of 0.18 nm and 0.25 nm, which are assigned to graphitic (102) and (020) planes respectively, were scarcer. It is important to highlight that the diagnostic graphitic basal planar (002) spacings of 0.31 nm were only observed in sample **5**. This indicates that under the reaction conditions used, the core morphology of **4** is less-ordered, with the long linker retarding graphitization.

The PL properties were also evaluated, and near-identical profiles were observed for **4** and **5**. The TTA-functionalised COOH-FCD **4** absorbance profile maxima were between 200–450 nm, which is characteristic for bottom-up synthesized FCDs. The defined peak at 270 nm can be attributed to π–π* transition of aromatic/alkenyl C=C bonds or C=N bonds (Fig. S4 in ESI)^45^. The fluorescence emission profile (λ_ex_ = 300–400 nm) exhibits the signature features of N-doped FCDs: blue-centred, excitation-dependent emission (Fig. 3C for **4**, and Fig. S5 in ESI for **5**). In addition, COOH-FCD **4** exhibited an optimal QY of 6% (for COOH-FCD **5**, QY = 4%), relative to quinine sulfate (Fig. S6). The excitation spectrum (λ_em_ = 420 nm) shows two peaks at 230 nm (π–π* of aromatic/alkenyl C=C bonds)^46^, and 340 nm (n–π* transitions in C=O/C=N bonds, Figs 3C and S5)^47^.No photobleaching was recorded for either **4** and **5** after 30 min of continuous irradiation in-solution (λ_ex_ = 340 nm, λ_em_ = 440 nm, Figs S7 and 8), whereas Rhodamine 6 G showed a 50% reduction over the same time-period. Stability was further examined by monitoring the emissive intensity at pH 0–14. 100% intensity was retained between pH 6–10, and >75% from pH 3–6 and 10–11 for both particles, see ESI for details. COOH-FCDs **4** and **5** were also exposed to increasing concentrations of NaCl in aqueous solution to test the effect of ionic strength (Fig. S10). At salt concentrations of up to 1 M, a 13% reduction of emission was observed in both nanoparticles (see Fig. S10 in ESI), demonstrating the PL robustness of the newly synthesised COOH-FCDs.

The chemical and functional group composition of COOH- FCDs **4** and **5** was determined by elemental analysis, Fourier-transformed infra-red spectroscopy (FTIR) and x-ray photoelectron spectroscopy (XPS). As expected, differences in C and O composition between **4** and **5** were also detected in their elemental composition (For **4**, C: 38.04, H: 8.15, N: 6.37, Cl: 7.31, O: 40.11; and **5** was C: 47.27, H: 7.68, N: 7.69, Cl: 5.40, O: 31.95; Table S1), which is in agreement with the different nature of the passivating linkers, with TTA-derived COOH-FCD containing a greater proportion of O atoms in their structure than β-alanine. Functional group analysis by FTIR did not show significant differences between both COOH-FCDs (Figs S11 and 12). Signature peaks for COOH-FCD **4** were observed for amino and/or alcohol groups (N-H/O-H) at 3360 cm^−1^. Signals at 2929 cm^−1^, C-H (sp^3^), were attributed to either the linker or to functional groups at the FCD surface. Amide groups (NHCO) at 1633 cm^−1^ indicated surface passivation, while a band centred at 1084 cm^−1^ could be assigned to C-O bonds of either the linker or residual carbohydrate. A large broad peak at 626 cm^−1^ indicated C-Cl bonds. A similar pattern was detected for **5**, see ESI Figs S11 and 12. XPS analyses gave near-identical results for both COOH-FCDs: the wide scan XPS spectrum for COOH-FCDs indicated the presence of C, O, N and Cl, in proportions similar to elemental analysis,with peaks at 285 eV (C 1s), 532 eV (O 1s), 400 eV (N 1s) and 197 eV (Cl 2p) for **4** (for **5** see Figs S13–16 ESI). High resolution scans of each peak were deconvoluted for functional group identification, and confirm the formation of aromatic C=O, -OH, and N-heteroaromatic moieties, as well as aliphatic C = O and C-Cl bonds (Tables S2 and 3).

The zeta potentials (electrokinetic potential) for COOH-FCDs **4** and **5** were in the range of −6.91 to +3.01 mV for **4**, and −6.73 to +2.29 mV for **5** (Figs S17 and 18). The values obtained in both cases are slightly more negative than previously produced FCD derived from diamine TTDA^39^, which can be attributed to an increase of carboxylic acids on the surface. This is further supported by the increase of lactose functionalization (between 7–10 times) found on lactose-functionalised FCDs **6** and **7** when compared to TTDA-derived lactose FCDs^39^ as determined by ^1^H-NMR qualitative analysis of the ratio between one of the lactose anomeric signals (δ4.57 ppm) and a proton shift assigned to the FCD core at δ5.07 ppm (see Fig. S27 and Table S4 in ESI), which suggests more acid groups are available for conjugation by the glycan biomolecule. Furthermore, these results also indicate a complex composition of surface functional groups, irrespective of linker length. Both COOH-FCD solubilities in aqueous media were excellent up to 20 mg mL^−1^. Solutions exposed to light at room temperature for several months were stable. Structural characterization by NMR (Figs S19 and 20) revealed specific signals associated with the surface linkers: in the ^1^H NMR of **4**, proton signals at δ 1.65, 1.83, 2.50, 2.98, 3.12, 3.43 and 3.45–3.61 ppm; in **5** proton signals at δ 2.65 and 3.15 ppm associated to the β-alanine short linker. The presence of carboxylic acid surface functionality was further evidenced by the asymmetric nature of the conjugated linker highlighted in the ^1^H-NMR - e.g. for **5**, the signature triplet splitting pattern assigned to the two central methylene groups in the β-alanine linker at δ 2.65 and 3.15 ppm, suggesting two different functional groups at either end (e.g. amide and acid groups) (Figs S19 and 20). In addition, two similar sets of signals with different intensity levels, suggested the presence of various organic species with lower intensity proton shifts between δ 2.50–4.50 ppm being assigned to surface-bound polyhydroxylated architectures. ^1^H-^13^C HMBC NMR analysis of these peaks revealed correlations with cross-peaks at 170–185 ppm signifying closely connected amorphous/sp^3^ and sp^2^-surface domains (Figs S21 and 22). ^1^H NMR, identified conserved and well-resolved singlets at σ 8.38, 8.56 and 5.00 ppm in both COOH-FCD **4** and **5**. ^1^H-^13^C HSQC and HMBC NMR experiments further uncovered the likely molecular structure of these conserved signals, (Figs S21 and 24). One-bond correlations between proton shifts at δ 5.00 ppm and ^13^C signals at 70 ppm and 2–3 bond connectivity to aromatic carbon shifts at σ 142 and 154 ppm were also unveiled, which can be assigned to pyrazine motifs. Moreover, singlets at δ 8.38 and 8.56 ppm, associated to *N*-heteroaromatic species^47^, showed direct connectivity to aromatic carbon signals at 144 and 142 ppm, respectively, and 2–3 bond correlations to a signal at 154 ppm. These results indicate the presence of simple N-heteroaromatic species, likely a pyrazine-type motif^48^.1,2-aminoaldoses, which we have identified as a key intermediate in the synthesis of FCDs from glucosamine^39^, can self-dimerise in a sequential cyclisation/condensation reaction to afford pyrazine rings with polyhydroxyl arms derived from the remaining carbohydrate moeity^49–52^. Indeed, the formation of fructosazine, a polyhydroxylpyrazine from the browning of glucosamine is a well-studied reaction^53^. Thus, we suspect a similar polyhydroxylpyrazine as a common motif in both COOH-FCDs **4** and **5**.

### Mechanism of COOH-FCD Formation

**Figure 4:**
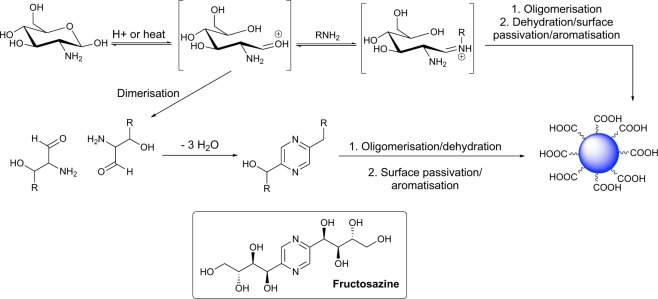
Proposed mechanism of COOH-FCD formation.

In order to ascertain the reaction mechanism of the formation of COOH-FCDs, a time-point ^1^H and ^13^C NMR analysis of the reaction was carried out at 30 s intervals up to 180 s. β-alanine-derived FCD **5** was chosen as the model system, as both FCDs exhibit the same conserved signals in NMR spectra (Figs S28 and 29). Although pyrazine-type signals (δ 8.40 and 8.55 ppm) start appearing slowly at 30 s of MW heating, significant formation occurs only after 120 s. Carbohydrate anomeric signals δ 4.60–5.30 ppm, assigned to the ring-closed pyranose, also undergo significant change after 120 s. This transition, after 2 minutes of MW irradiation, is also evident in the ^13^C NMR spectra: loss of the anomeric carbon at σ 90 ppm, increased complexity of aromatic-associated signals (σ 140–180 ppm) and variations in signals at δ 20–80 ppm; the latter assigned to the pyranose ring (Fig. S29). Moreover, after 120 s MW heating, the characteristic carbonyl C=O signal from an acid functional group was observed in the ^13^C spectra at δ 177 pm signal, further confirming the presence of acid groups on the FCD surface. After the first 120 s, loss of solvent leads to a critical concentration of precursors, in which subsequent carbonisation and aromatisation can occur, leading to the formation of the pyrazine-type motif (Fig. 4 and S1 in ESI). Interestingly, the PL properties of **5** measured at different reaction times (from 30 to 150 s) did not significantly change the emission profile with the exception emission at ex = 300 nm, which was optimum at 120 s. These results suggest that fluorophores contributing to the PL properties of the material are generated early on in the synthesis with an optimum profile at 120 s (See Figure S30 in ESI).

### Fe^3+^ Sensing Studies

The development of fast and cheap technologies to detect Fe^3+^ species is of great interest for monitoring water quality and for understanding biological processes^54,55^. The redox properties of Fe^3+^ are part of important physiological processes^55^. Qualitative and quantitative Fe^3+^ detection can be achieved by voltammetry^56,57^, atomic absorption spectrometry^58^, and inductively coupled plasma mass spectrometric (ICP-MS) methods^59^. Most detection methods use emissive species such as organic dyes^60^, QDs^61^, and metal-organic frameworks^62–65^, which are quenched in the presence of Fe^3+^. However, these materials are often expensive and protocols to access them are labour-intensive. Moreover, most fluorophores are often not temperature, light or air-photostable. The use of water soluble FCDs as heavy metal sensors for Hg^2+^, Fe^3+^, Cr^2+^ has been described in recent years^20^, although little is known about the sensing mechanisms.

**Figure 5:**
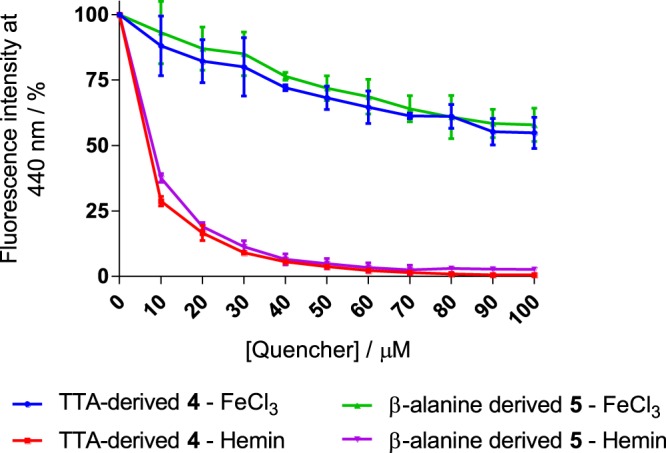
Comparison of quenching with Fe^3+^ and hemin of **4** and **5** in deionised water.

In addition to pyrazine-type moieties, we also identified the presence of polyhydroxyl aromatics in the surface of COOH-FCDs **4** and **5**. We thus hypothesised that COOH-FCDs could act as selective sensors for Fe^3+^ in a similar fashion to catechol-siderophores: 1,2-hydroxybenze motifs (catechols) can strongly chelate to Fe^3+^ due to a hard-hard interaction, as defined by Hard-Soft Acid-Base (HSAB) theory^66^. For instance, enterobactin, a common siderophore in mainly Gram-negative bacteria, uses several catechol motifs to selectively scavenge Fe^3+^ over Fe^2+ ^^67^. To explore this application, 500 µM solutions of metal chloride salts of Fe^3+^, Fe^2+^, Ca^2+^, Cu^+^, Cu^2+^, Mn^2+^, and Mg^2+^, in addition to a wide range of salts containing those metals, were exposed to FCD **4** in deionized (dd)-water (see Fig. S32 in ESI). While minimal fluorescence depletion was observed with all tested salts, significant quenching (around 91%) was observed for Fe^3+^. Fe^2+^ only quenched the FCD fluorescence by 15%, which implies siderophore-type selectivity. Next, the effect of linker length and atomic nature on COOH-FCDs **4** and **5** towards Fe^3+^ sensing was evaluated in a Fe^3+^ titration experiment (0–500 µM in dd-water, Fig. S33). Overall, a 10% decrease in fluorescent quenching was observed for COOH-FCD **4**, which bears a longer linker. This result highlights the requirement for interactions between the Fe^3+^ and the polyhydroxyl aromatics and sp^2^ surface domains in the COOH-FCD. The steric bulk of the linker appears to interfere by somewhat blocking the surface emissive centres. To explore the selectivity for Fe^3+^ detection in a biologically relevant molecule, hemin, which contains a ferric cation surrounded by a protoporphyrin ligand, was examined. Hemin fluorescence quenching of **4** and **5** was superior to that for Fe^3+^ (Fig. 5). For example, at 10 µM, a 10% decrease was measured for Fe^3+^ and a 73% decrease in hemin emission was observed, 95% of the FCD fluorescence was quenched when COOH-FCDs **5** was exposed to 40 µM hemin. We attribute the enhanced quenching effect for hemin to the presence of the porphyrin motif, which allows for additional π-π stacking interactions to the COOH-FCD surface sp^2^-domains.

### Acid coated-FCDs for live cell imaging

The biocompatibility of COOH-FCDs with cells is an absolute requirement for their use in live cell imaging applications. To that end, cell toxicity and internalisation studies were conducted in MDA-MB-231 (human breast) cancer cells. MDA cultures were exposed to concentrations from 10^−3^ to 2000 µgmL^−1^ for 1 hour, 1 and 3 days. Reductive metabolic (RM) competence was assessed by Alamar Blue, and the number of live cells with Calcein AM. Neither long linker COOH-FCD **4** or β-alanine-derived COOH-FCD **5** caused significant cell death at any exposure, duration and concentrations tested (Figs S34 and S35). RM alterations were measured after 1 h exposure at all tested concentrations. RM decreased to 60% after 3 days of continuous exposure at 2000 µgmL^−1^, highlighting interactions between FCD and cells. For either COOH-FCD LC_50_ was not reached even at the highest concentration and longest exposure.

**Figure 6:**
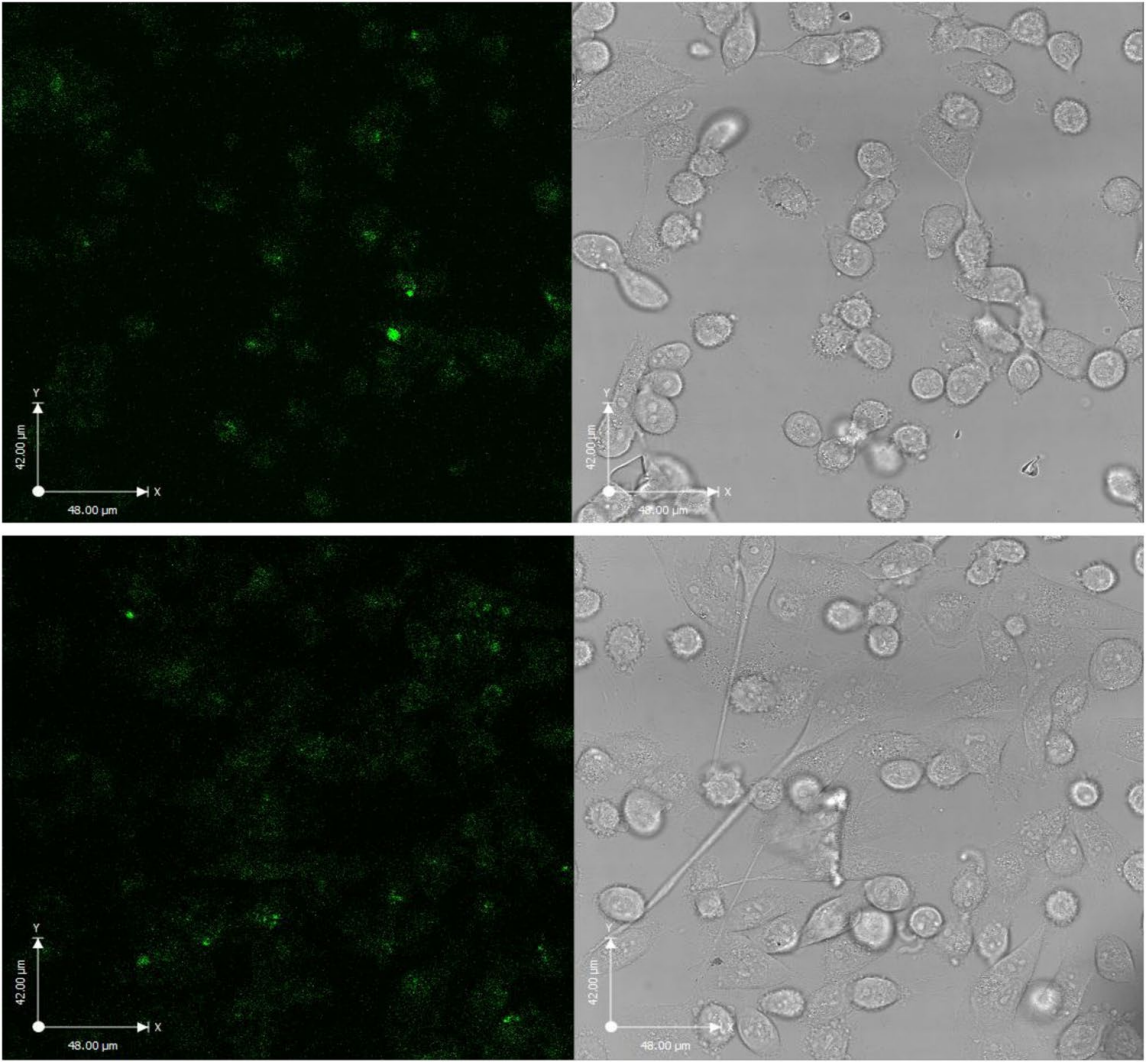
Confocal microscopy images of COOH-FCD **5** internalization in MDA cells exposed to 500 µgmL^−1^ for 2 h: (Top) 4 °C and (Bottom) 37 °C.

Confocal microscopy was used to monitor cellular uptake, evaluated as fluorescence per unit area (FPUA). In live MDA cells after 2 h exposure to 500 µgmL^−1^ of both COOH-FCDs at 4 °C and 37 °C (Fig. 6), there were no FPUA differences (Fig. S36), suggesting passive transport, without energy input, as internalization pathways for either FCD (for complete set of images including images of untreated cells see S37–39).

## Conclusion

In summary, we have shown the one-pot three-minute synthesis of carboxylic-acid FCDs (COOH-FCDs) from cheap and easily-accessible starting materials. The different COOH-FCDs bear similar surface functional groups, structural and PL properties, while the core structure, however, depends on the passivating agent. *β*-alanine-derived FCDs (**5)** result in more crystalline graphitic (sp^2^) cores, whereas TTA-linkers, which are longer and bear a higher O content, yield FCDs (**4**) with less ordered cores. We also show that the CDs are stable in a range of pH from 3–11 without significant loss of fluorescence. Moreover, the CDs were stable to purification on silica gel column and size-exclusion chromatography (G10 Sephadex). Control over the core morphology, provides unique opportunities to probe PL mechanisms as well as the tribological properties of these novel materials. A key pyrazine-type motif, which is likely generated from the self-dimerisation of the 1,2-aminoaldose intermediate, leads to disubstituted polyhydroxyl pyrazine motifs, e.g. fructosazine. The latter, upon continued heating and dehydration leads to FCD formation. We show that the novel COOH-FCDs can selectively detect Fe^3+^ and hemin in deionised water, which further supports the presence of the identified surface functional groups. Finally, we demonstrate that COOH-FCDs are readily internalised in MDA-MB-231 cancer cells via a passive uptake pathway and that the FCDs are non-toxic at high concentrations and very long continuous exposures.

## Electronic supplementary material

Supplementary information
