# Supplementary material for: Practical Three-Minute Synthesis of Acid-Coated Fluorescent Carbon Dots with Tuneable Core Structure
Source: Sci Rep. 2018 Aug 15;8:12234. doi: 10.1038/s41598-018-29674-2 (PMC6093873; doi:10.1038/s41598-018-29674-2)
Supplement: Supplementary file 1 — Supplementary information [file 41598_2018_29674_MOESM1_ESM.pdf]

Appendix A: Supplementary Information

Practical three-minute synthesis of acid-coated fluorescent carbon dots with tuneable core structure

Stephen A. Hill,[a] David Benito-Alifonso,[a] Sean A. Davis,[a] David J. Morgan,[b] Monica Berry,[a] and M. Carmen Galan[a*]

[a]School of Chemistry, University of Bristol, Cantock's Close, Bristol, BS8 1TS, UK.

[b]Cardiff Catalysis Institute, School of Chemistry, Cardiff University, Park Place, Cardiff, CF10 3AT, UK.

A)

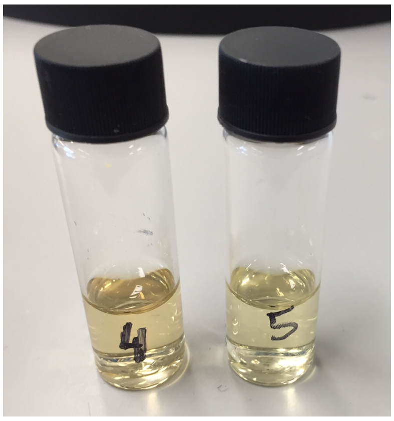

B)

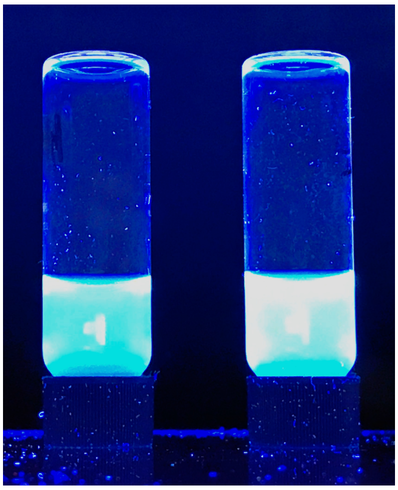

Figure S1. Photograph of FCD **4** (left) and **5** (right) under: A) natural light and B) under UV irradiation.

COOH-FCD Characterisation

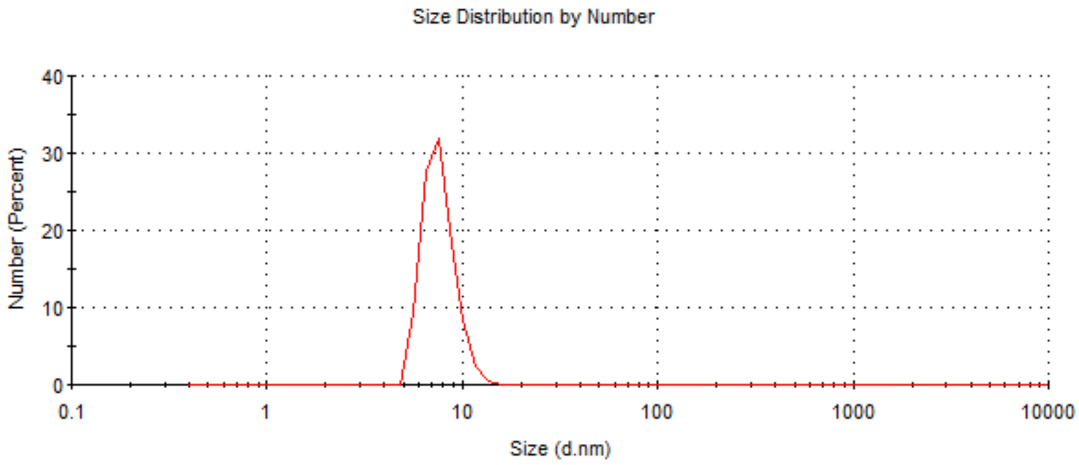

Figure S2A: DLS profile of **5** with a hydrodynamic diameter from 5-11 nm

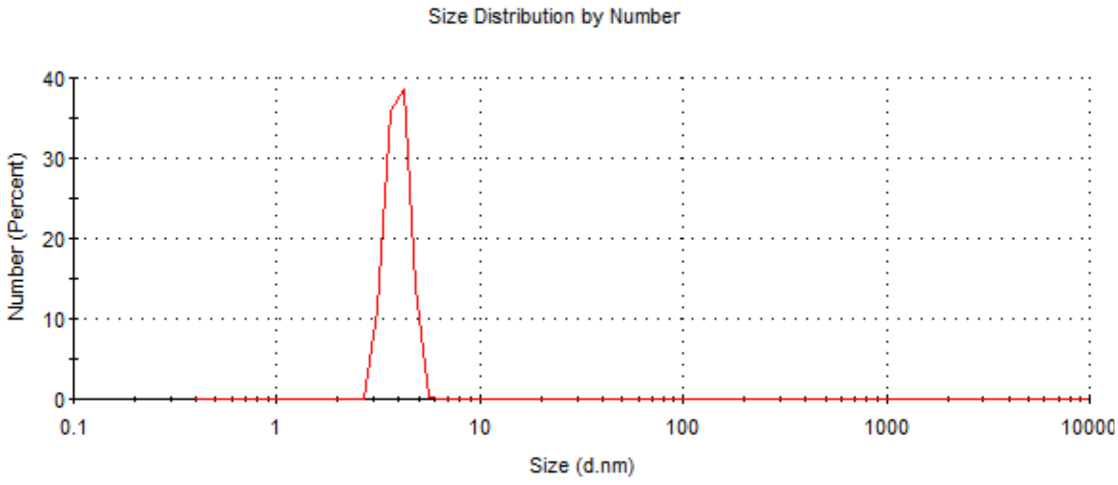

Figure S2B: DLS profile of **4** with a hydrodynamic diameter from 2-6 nm

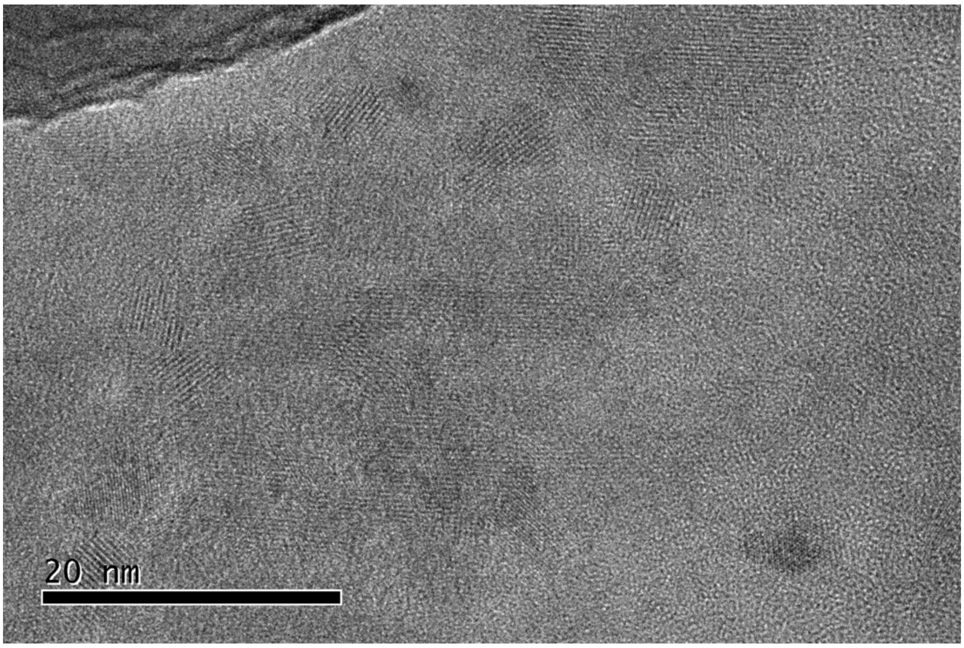

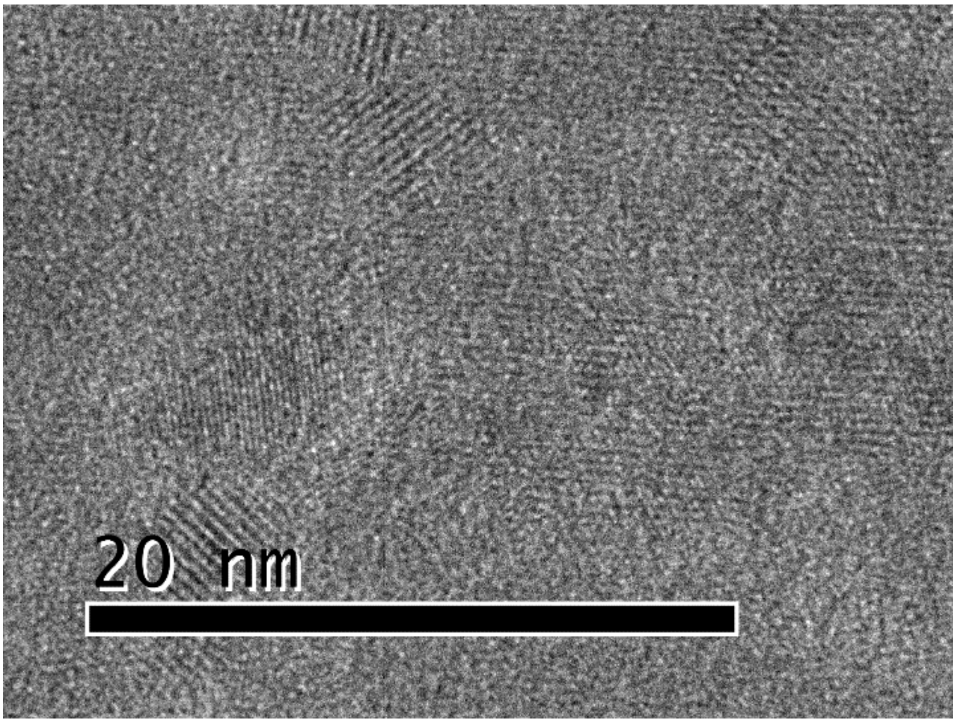

Figure S3A. Ilustrative HR-TEM images and sizing of β-alanine-derived FCD **5**

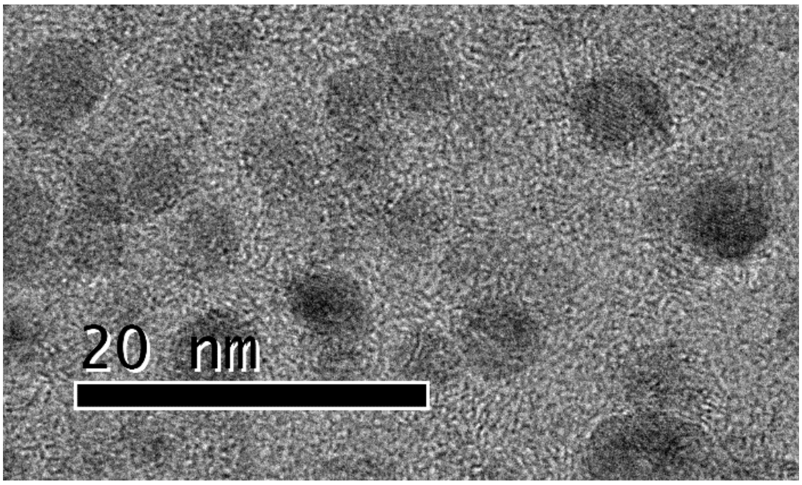

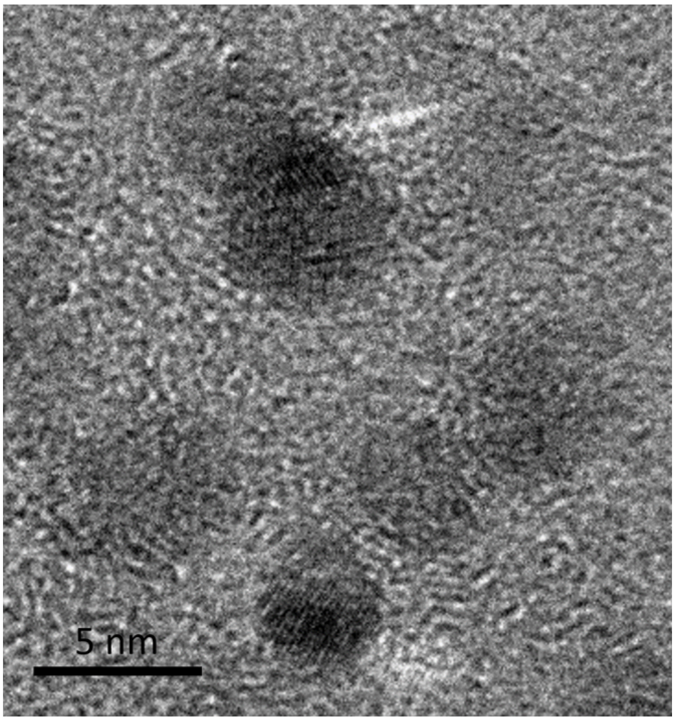

Figure S3B. Ilustrative HR-TEM images and sizing of FCD **4**

A)

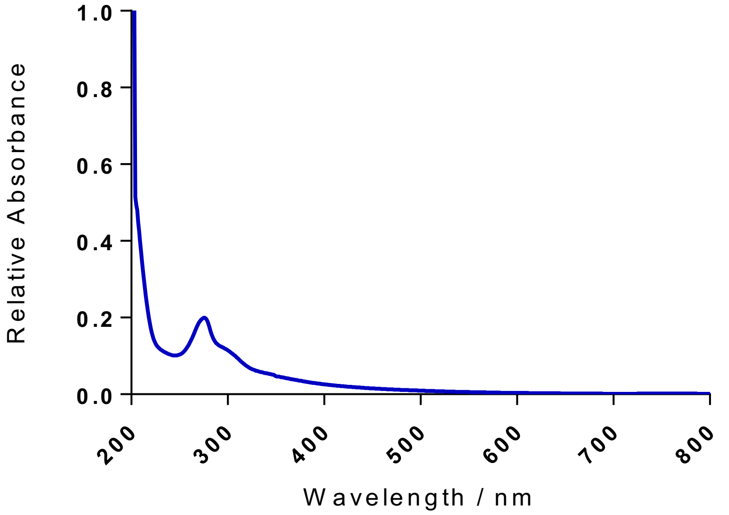

B)

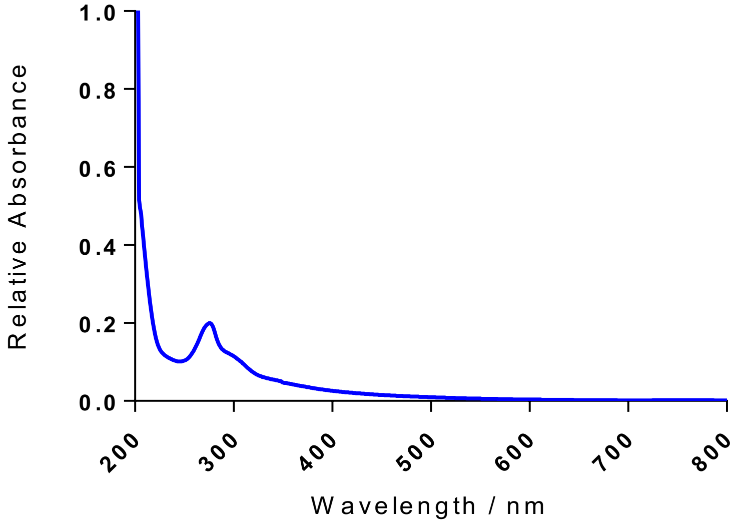

Figure S4 UV-vis absorbance spectrum of COOH-FCD A) **4** and B) **5**

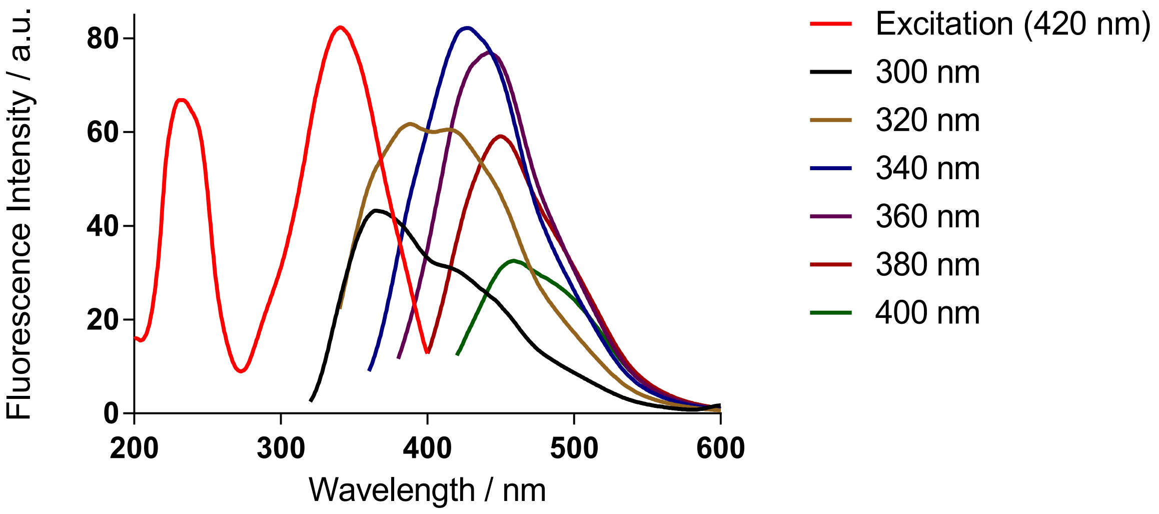

Figure S5: Fluorescence excitation and emission spectrum of COOH-FCD **5**

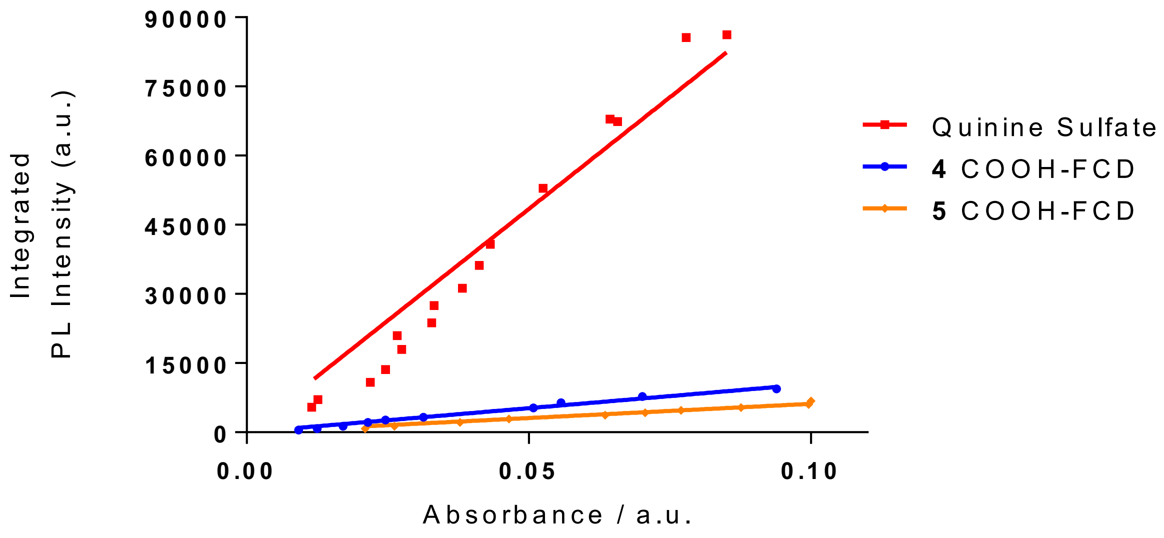

Figure S6: Quantum yield of fluorescence for COOH-FCDs **4** and **5**.

The quantum yield of fluorescence (QY or Φ) for **4** and **5**, in aqueous media (refractive index; η= 1.33) was calculated by measuring the integrated fluorescence intensity (excitation 340 nm) at a range of absorbance values below 0.10. The resulting curve was plotted and compared against quinine sulfate (QS) in 0.1M H_2SO_4 (refractive index; η= 1.33), which is a standard of known QY of 54%. (I = slope η = refractive index)

$$\Phi_{FCD} = \Phi_S \ x \left(\frac{I_{FCD}}{I_{QS}}\right) x \left(\frac{{\eta_{FCD}}^2}{{\eta_{QS}}^2}\right)$$

$$\Phi_{Long\ Linker\ COOH-FCD\ \mathbf{4}} = 0.54\ x \left(\frac{104690}{968694}\right) x \left(\frac{1.33^2}{1.33^2}\right) = 0.06$$

$$\Phi_{Short\ Linker\ COOH-FCD\ \mathbf{5}} = 0.54\ x \left(\frac{78428}{968694}\right) x \left(\frac{1.33^2}{1.33^2}\right) = 0.04$$

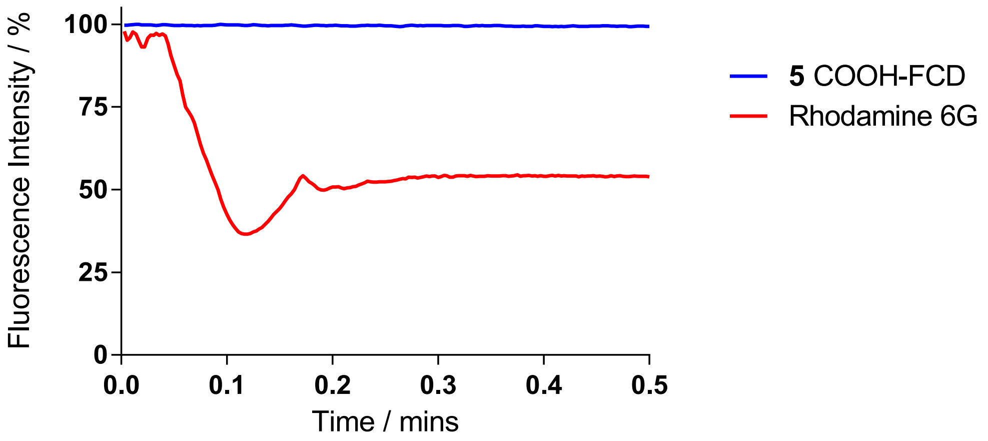

Figure S7: Fluorescence emission intensity of COOH-FCD **5** (λ_{ex} 340 nm, λ_{em} 440 nm) and Rhodamine 6G (λ_{ex} 360 nm, λ_{em} 600 nm) with continuous irradiation

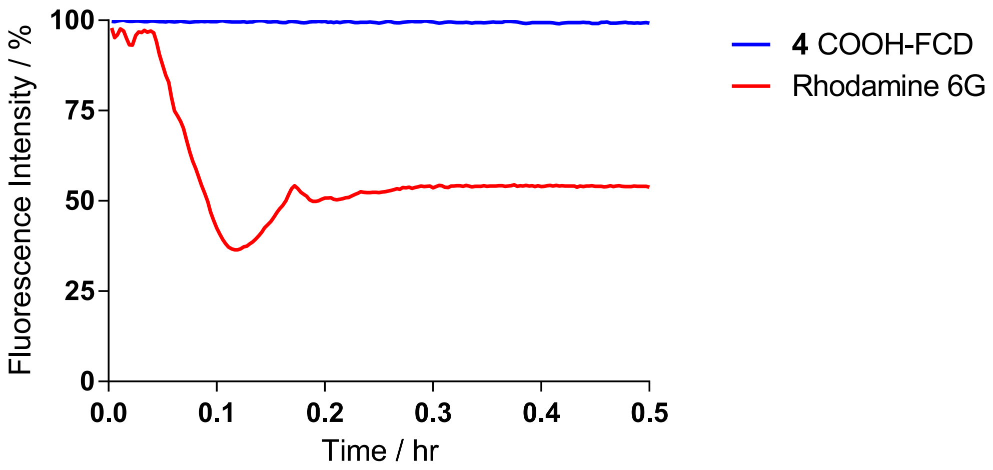

Figure S8: Fluorescence emission intensity of TTA-derived COOH-FCD **4** (λ_{ex} = 340 nm, λ_{em} = 440 nm) and Rhodamine 6G (λ_{ex} 360 nm, λ_{em} 600 nm) with continuous irradiation

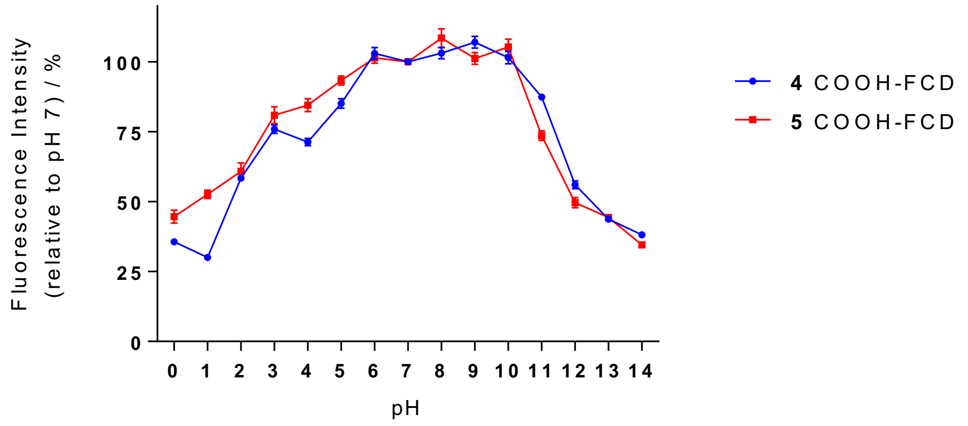

Figure S9: Fluorescence emission intensity of **4** and **5** (λ_{ex}= 340 nm, λ_{em}= 440 nm) as a function of pH. Fluorescence is presented as percentage of output at pH 7

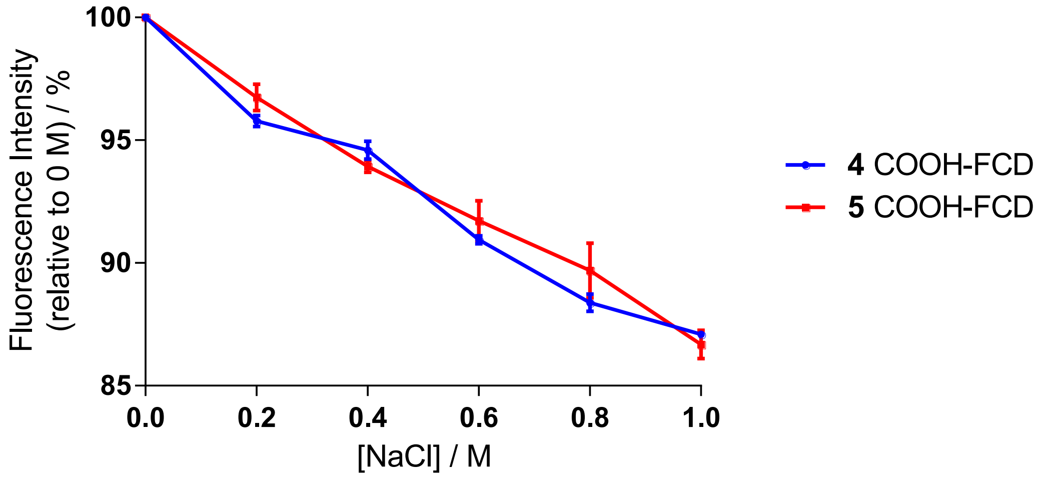

Figure S10: Fluorescence emission intensity of **4** and **5** (λ_{ex}= 340 nm, λ_{em}= 440 nm) as a function of ionic strengths. Fluorescence is presented relative to 0M

Table S1: Elemental composition of COOH-FCD **4** and **5**

COOH-FCD 4	C	H	N	Cl	O
**1**	42.86	7.79	7.16	8.47	33.70
**2**	38.44	8.22	6.45	7.19	39.68
**3**	32.83	8.44	5.48	6.28	46.95
**Average**	**38.04**	**8.15**	**6.37**	**7.31**	**40.11**

COOH-FCD 5	C	H	N	Cl	O
**1**	50.12	7.37	8.56	5.69	28.24
**2**	45.14	7.46	7.58	6.25	33.54
**3**	46.54	8.21	6.92	4.25	34.06
**Average**	**47.27**	**7.68**	**7.69**	**5.4**	**31.95**

The differences found between batches are due to small differences between samples and also are within the standard error of the measurement.

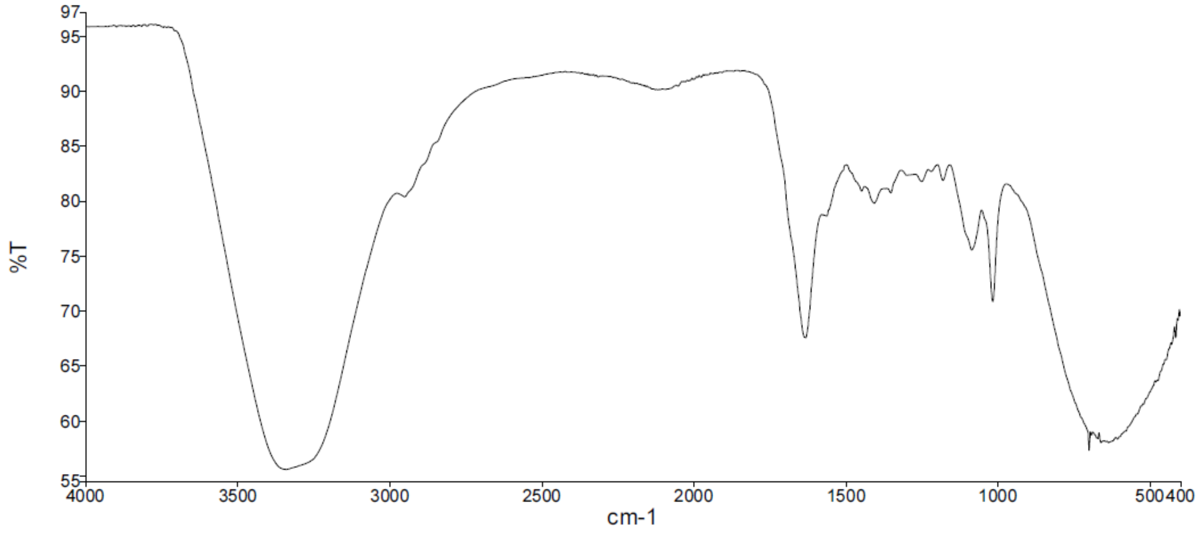

Figure S11: FTIR spectrum of COOH-FCD **4**; Key features: 3360 cm^{-1} (N-H/O-H); 2929 cm^{-1} (C-H (sp^3)); 1633 cm^{-1} (NHCO group); 1084 cm^{-1} (C–O); 626 cm^{-1} (C–Cl)

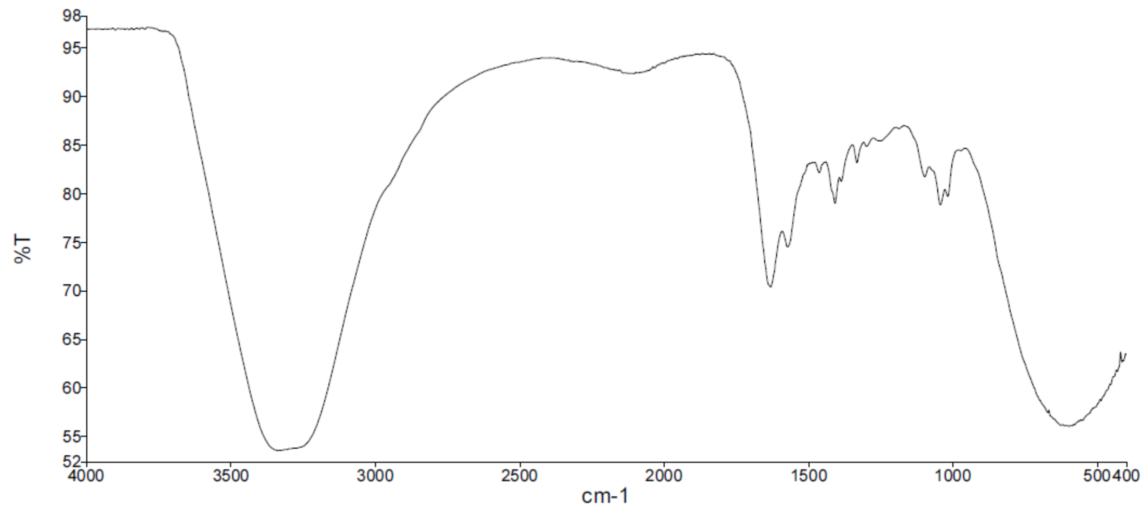

Figure S12: FTIR spectrum of COOH-FCD **5**; Key features: 3361 cm^{-1} (N-H/O-H); 2931 cm^{-1} (C-H (sp^3)); 1635 cm^{-1} (NHCO group); 1082 cm^{-1} (C–O); 625 cm^{-1} (C–Cl)

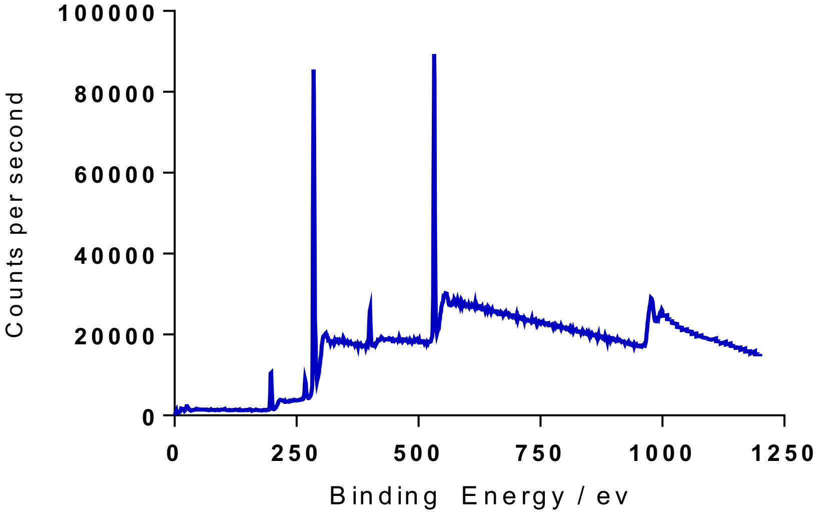

Figure S13: XPS spectrum of COOH-FCD **4**; Key Features: 285 eV (C 1s), 532 eV (O 1s), 400 eV (N 1s) and 197 eV (Cl 2p)

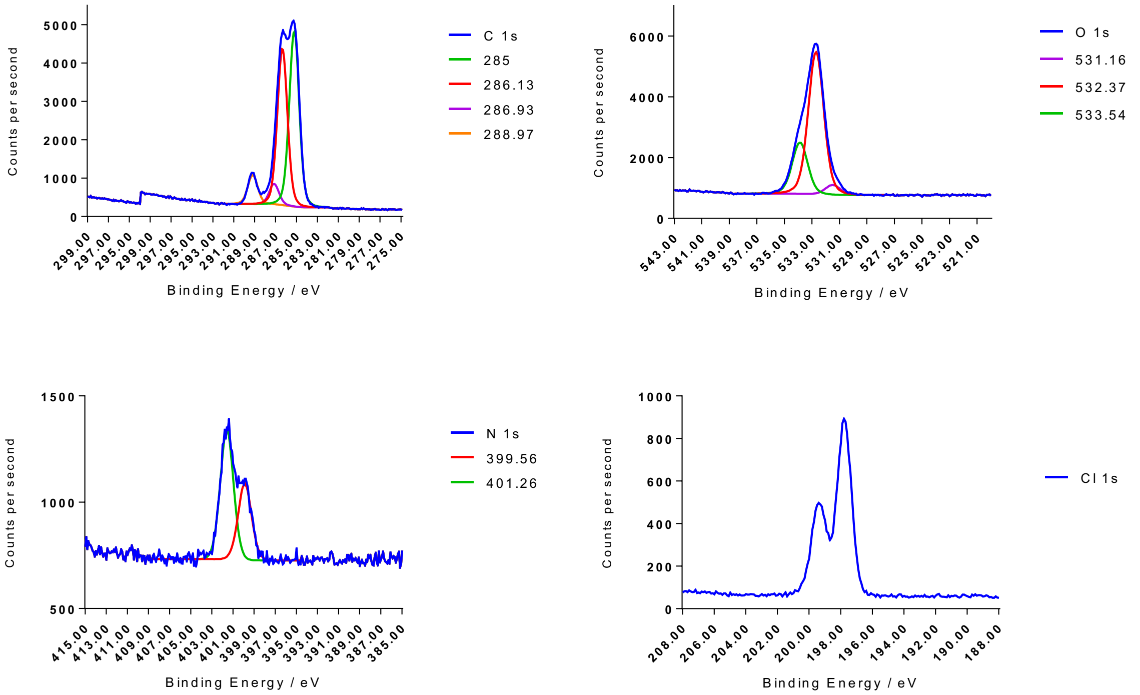

Figure S14: High-resolution XPS for C, O, N, and Cl plus deconvolutions (blue lines are raw data) for COOH-FCD **4**

Table S2: **4** - XPS deconvolution and functional group assignments

Element	Binding Energy Max / eV	Deconvolution Functionality
**C**	285.00	C-C
**C**	286.13	C-O-C/C-O or C-N
**C**	286.93	C=O
**C**	288.97	O-C=O (ester)
**O**	531.16	Aromatic C=O
**O**	532.37	Aliphatic C=O
**O**	533.34	Aromatic OH
**N**	399.56	NH_2 (amine or pyridine)
**N**	401.26	Imide/N-C=O
**Cl**	198.10	C-Cl
**Cl**	199.75	C-Cl

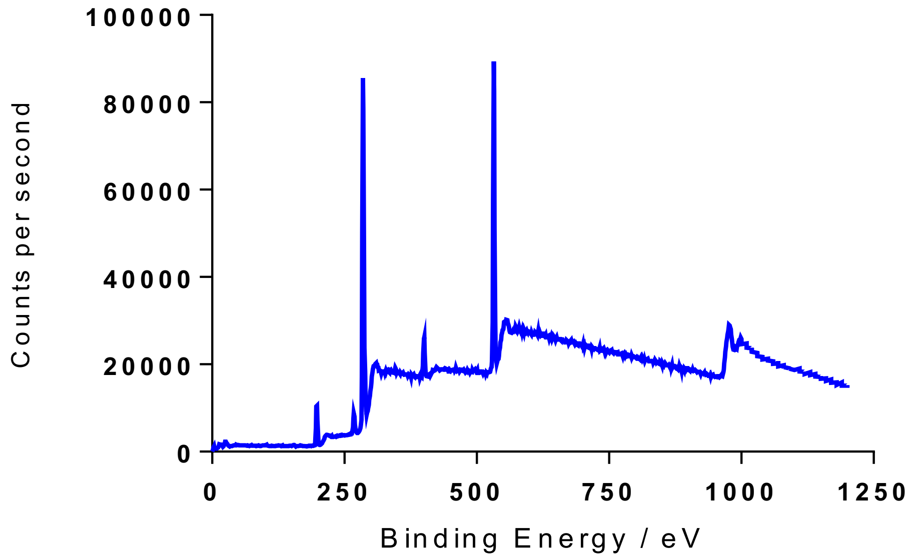

Figure S15: XPS spectrum of COOH-FCD **5**. Key Features: 285 eV (C 1s), 532 eV (O 1s), 400 eV (N 1s) and 197 eV (Cl 2p)

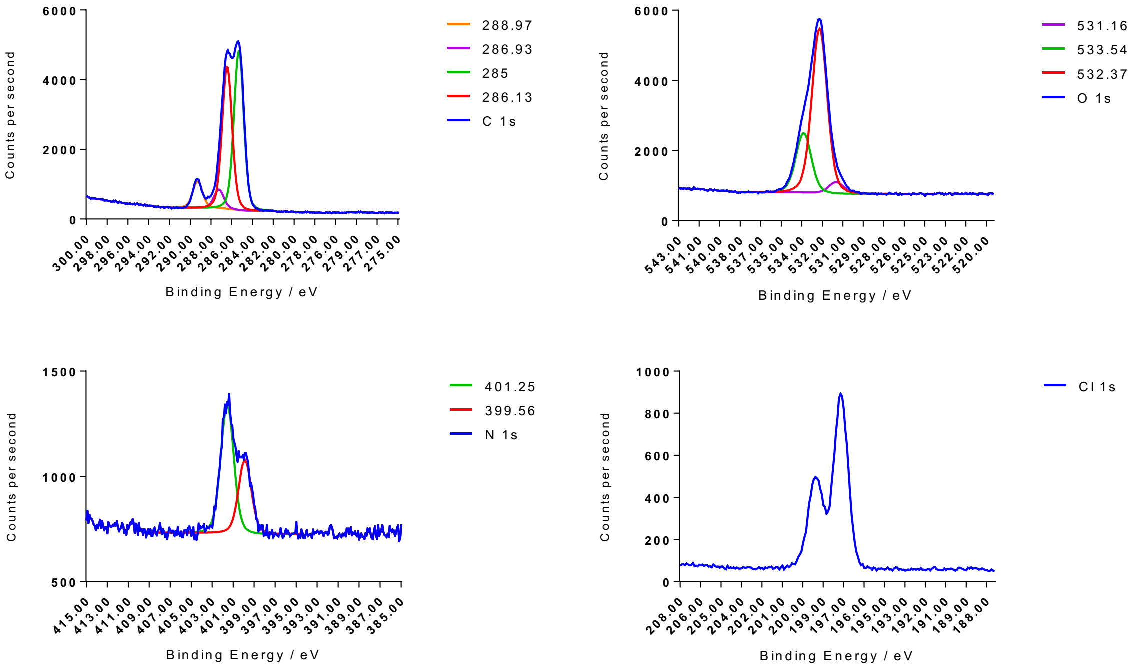

Figure S16: High-resolution XPS for C, O, N, and Cl plus deconvolutions (blue lines are raw data) of COOH-FCD **5**

Table S3: **5** - XPS deconvolution and functional group assignments

Element	Binding Energy Max / eV	Deconvolution Functionality
**C**	285.00	C-C
**C**	286.13	C-O-C/C-O or C-N
**C**	286.93	C=O
**C**	288.97	O-C=O (ester)
**O**	531.16	Aromatic C=O
**O**	532.37	Aliphatic C=O
**O**	533.54	Aromatic OH
**N**	399.56	NH_2 (amine or pyridine)
**N**	401.25	Imide/N-C=O
**Cl**	198.11	C-Cl
**Cl**	199.74	C-Cl

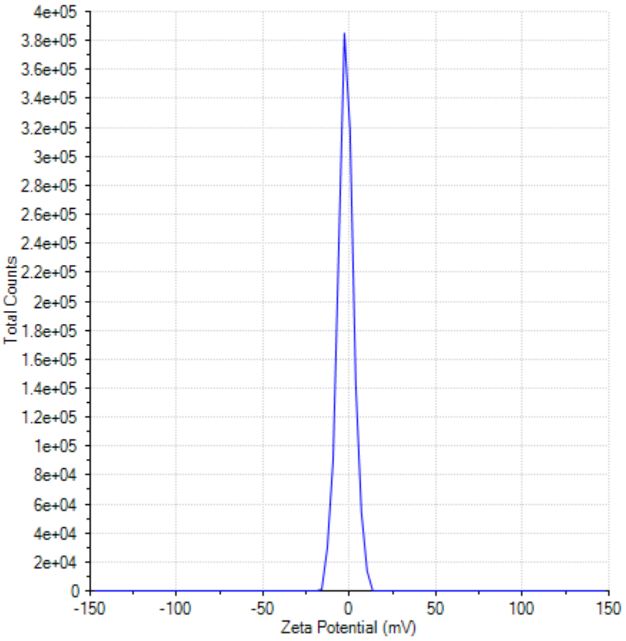

Figure S17: Zeta-potential of **4** (-6.91 to +3.01 mV)

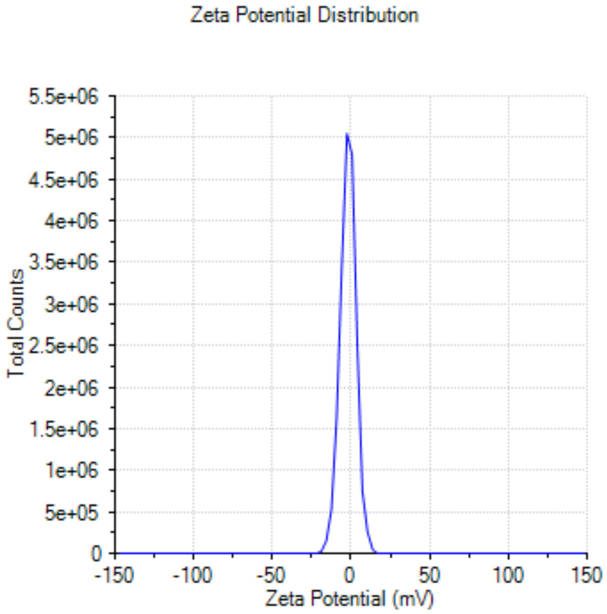

Figure S18: Zeta-potential of **5** (-6.73 to +2.29 mV)

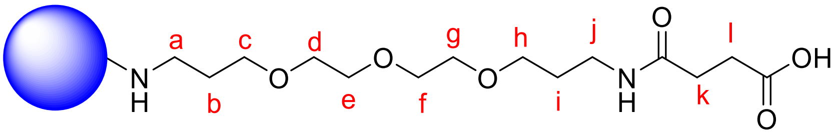

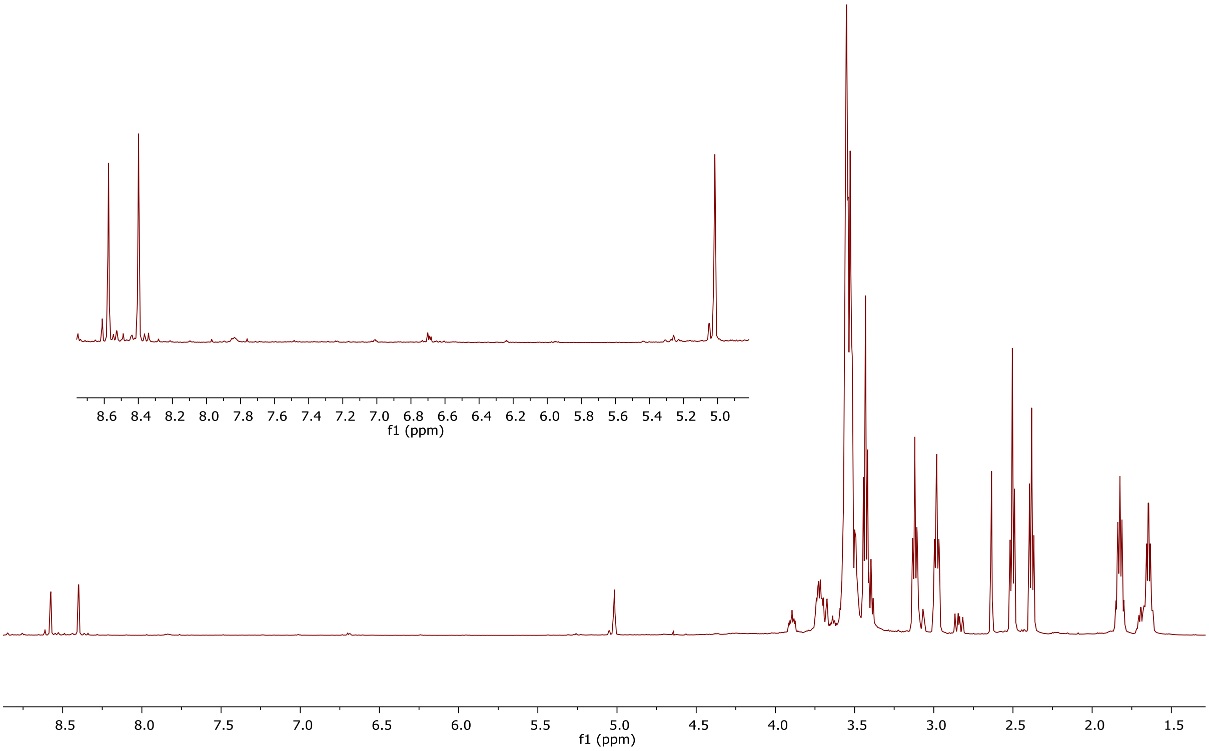

Figure S19: ^{1}H NMR spectra in D_2O of **4** (500 MHz, D_2O) δ: 3.61-3.45 (d-g), 3.43 (t, J = 10 Hz, c, h), 3.12 (t, J = 10 Hz, j), 2.98 (t, J = 10 Hz, a), 2.50 (t, J = 10 Hz, l), 2.38 (t, J = 10 Hz, k), 1.83 (p, J = 5 Hz, b), 1.65 (p, J = 5 Hz, i)

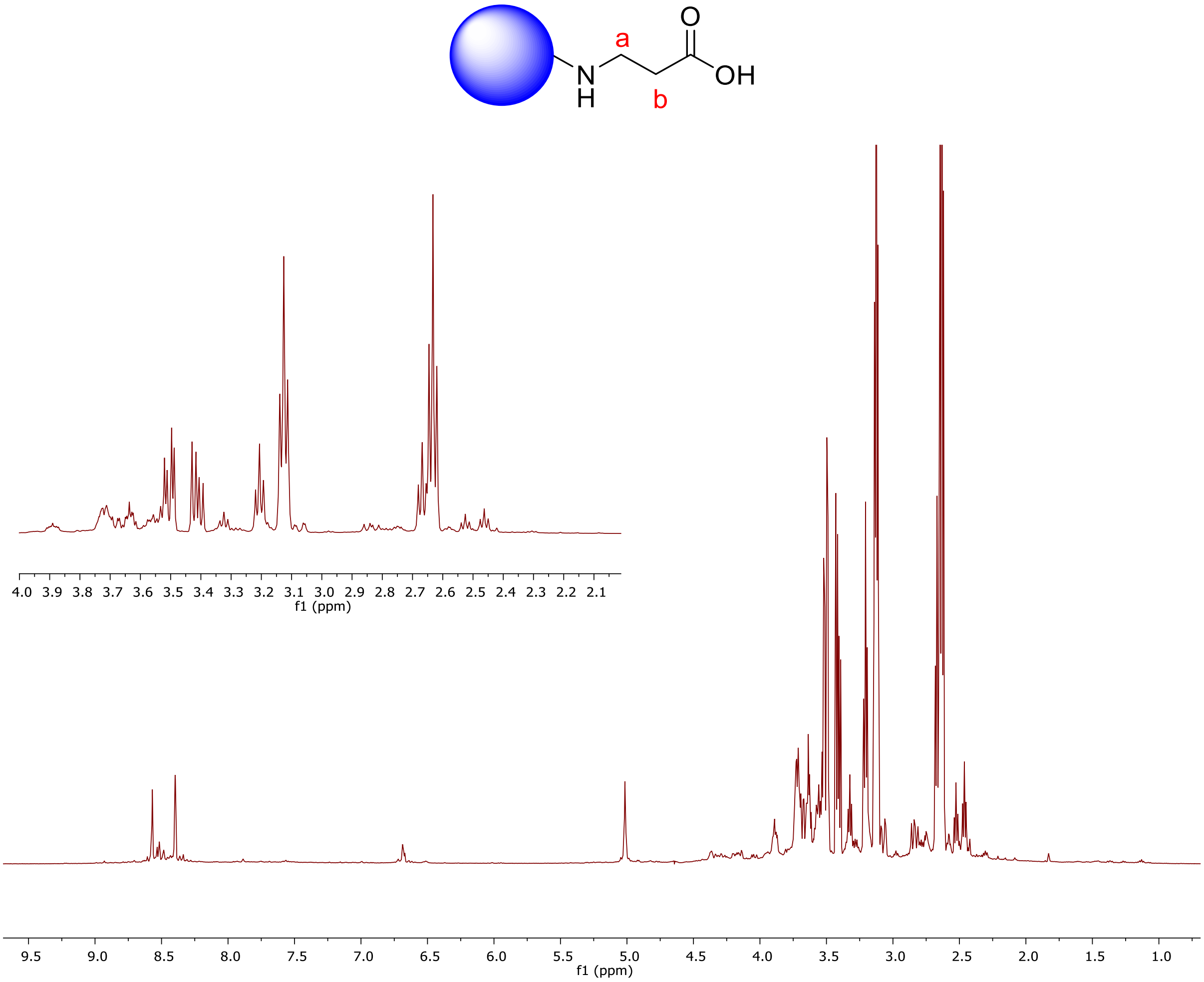

Figure S20: ^{1}H Presat NMR of **5** (500 MHz, D_2O) δ: 3.15 (t, J = 12 Hz, a) 2.65 (t, J = 12 Hz, b)

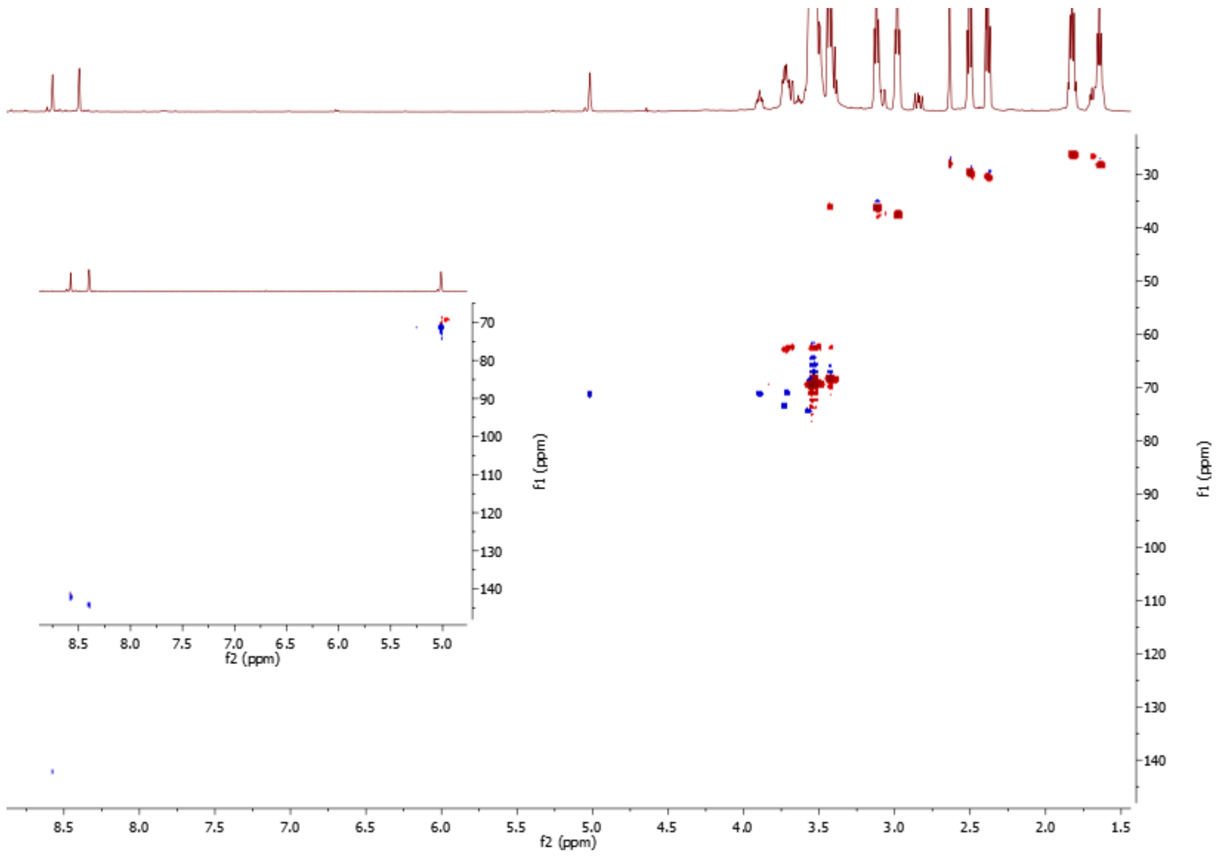

Figure S21: ^{1}H - ^{13}C HSQC of **4** (500 MHz, D_2O)

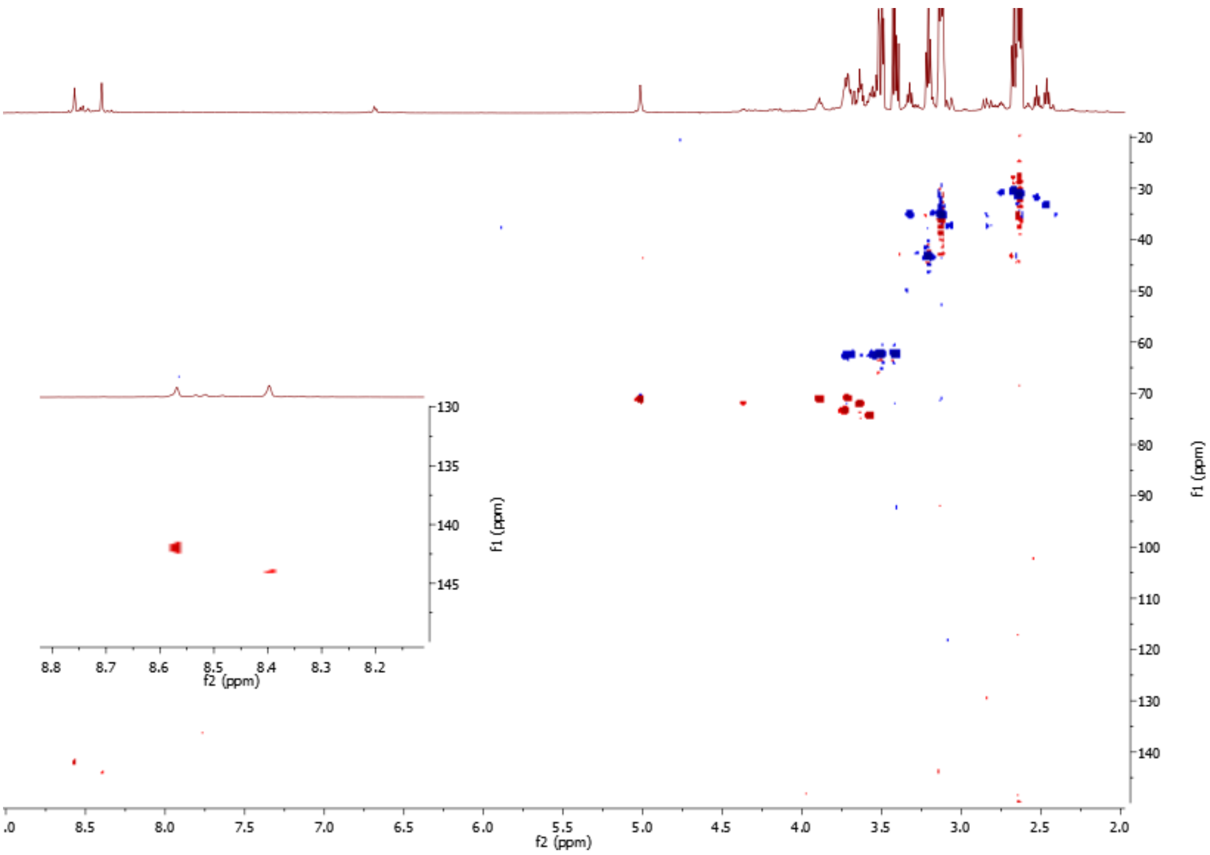

Figure S22: ^{1}H - ^{13}C HSQC of **5** (500 MHz, D_2O)

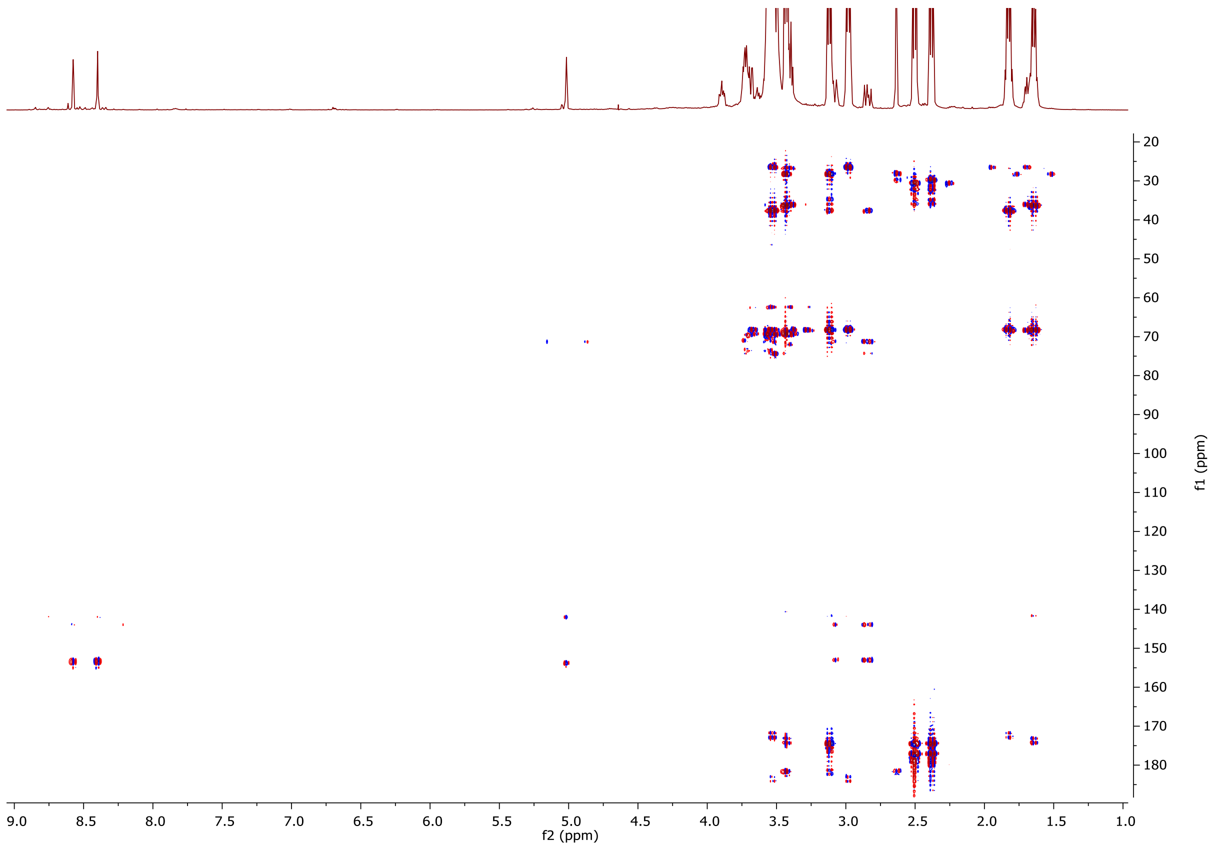

Figure S23: ^{1}H - ^{13}C HMBC of **4** (500 MHz, D_2O)

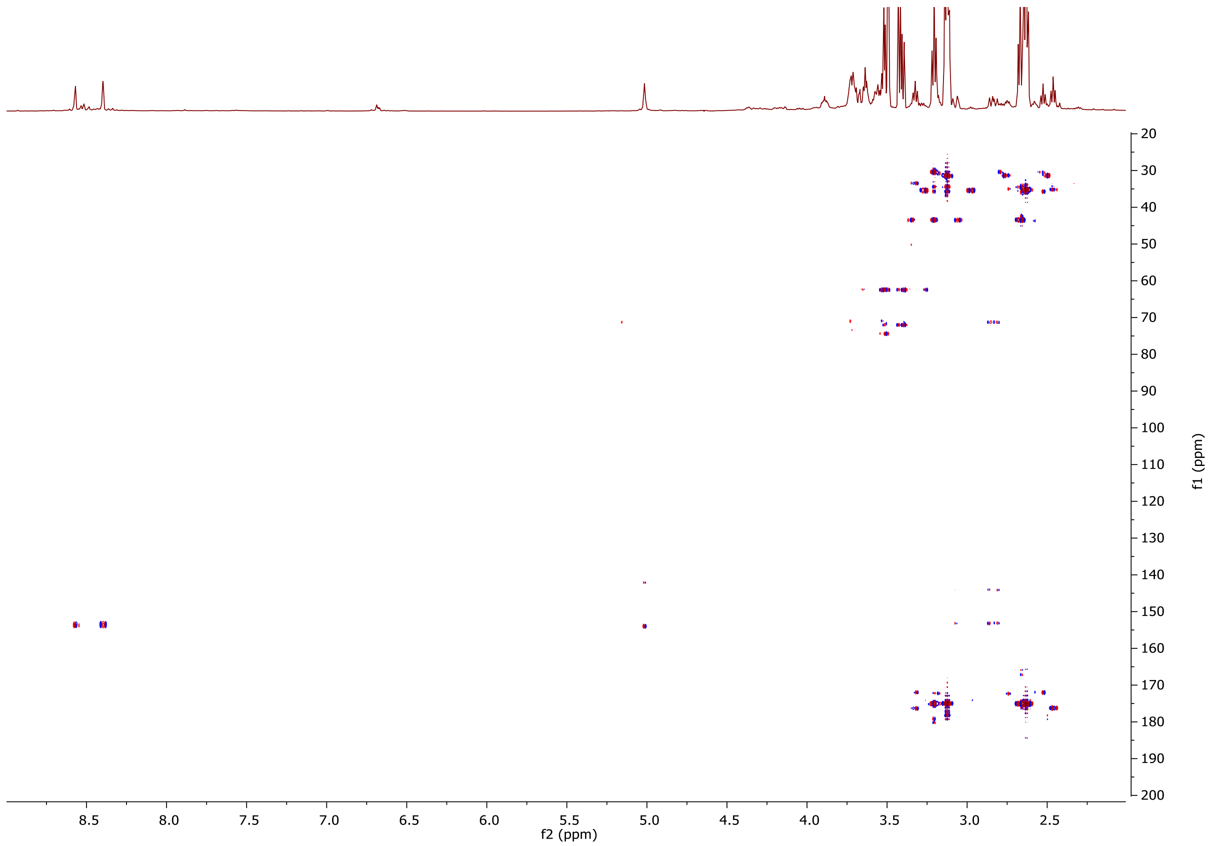

Figure S24: ^{1}H - ^{13}C HMBC of **5** (500 MHz, D_2O)

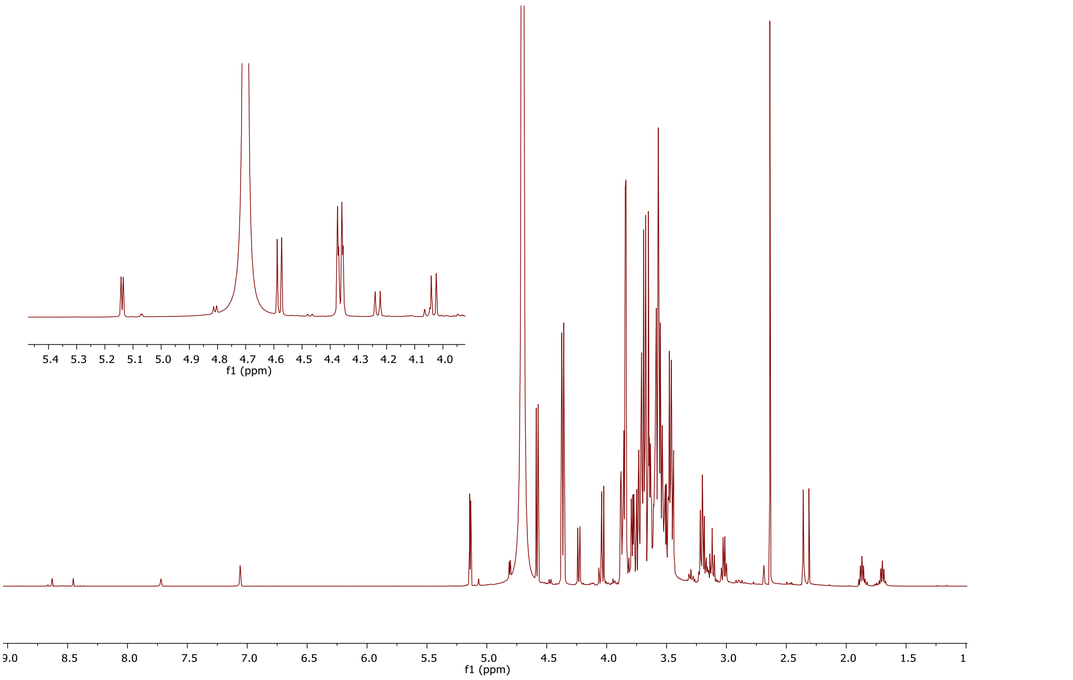

Figure S25: ^{1}H NMR of **6** - lactose-functionalised COOH-FCD **4** (D_2O, 500 MHz)

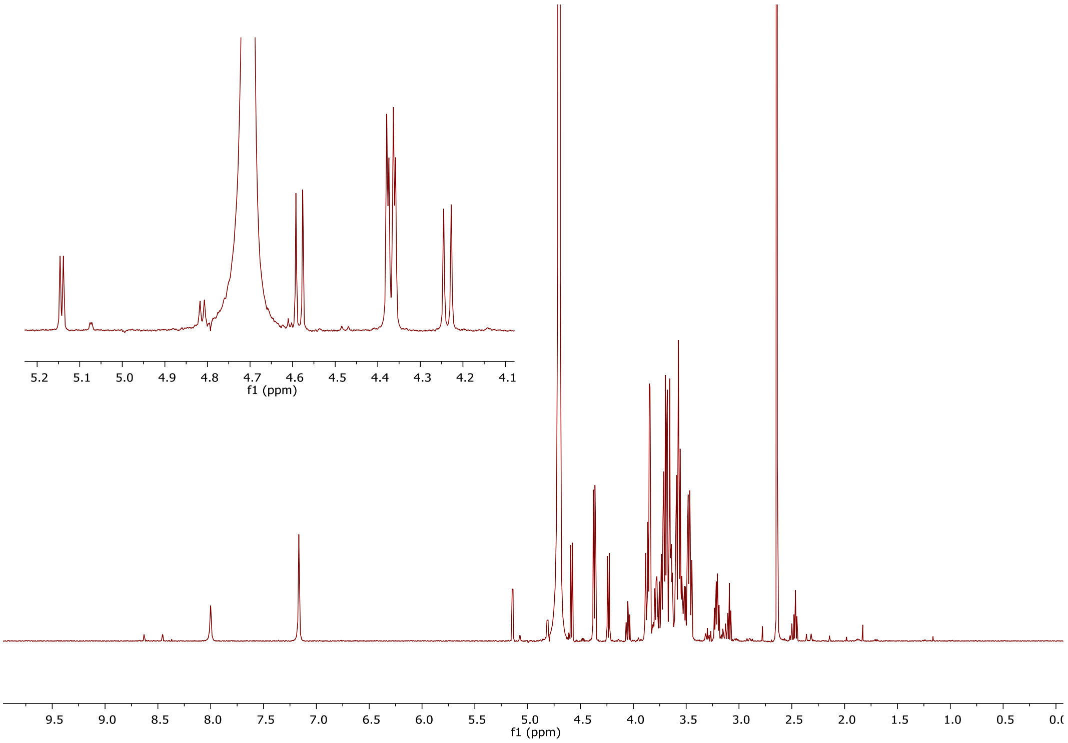

Figure S26: ^{1}H NMR of **7** - lactose-functionalised COOH-FCD **5** (D_2O, 500 MHz)

A)

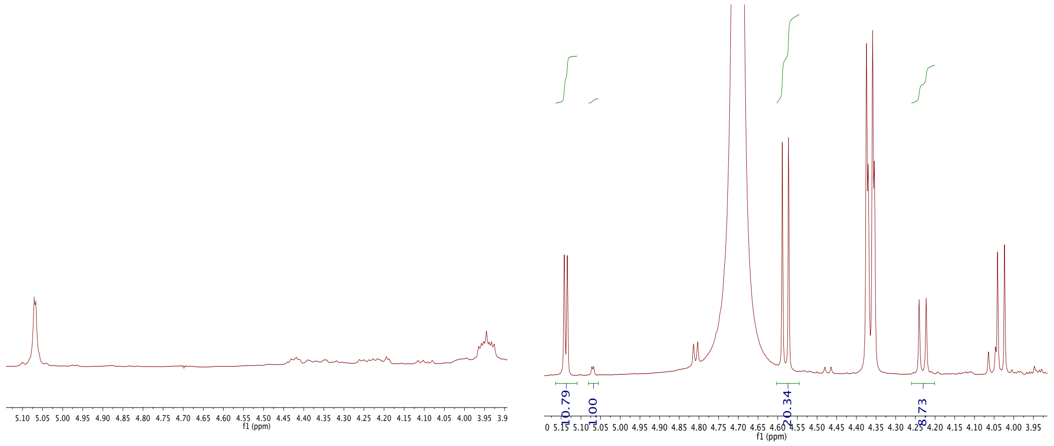

B)

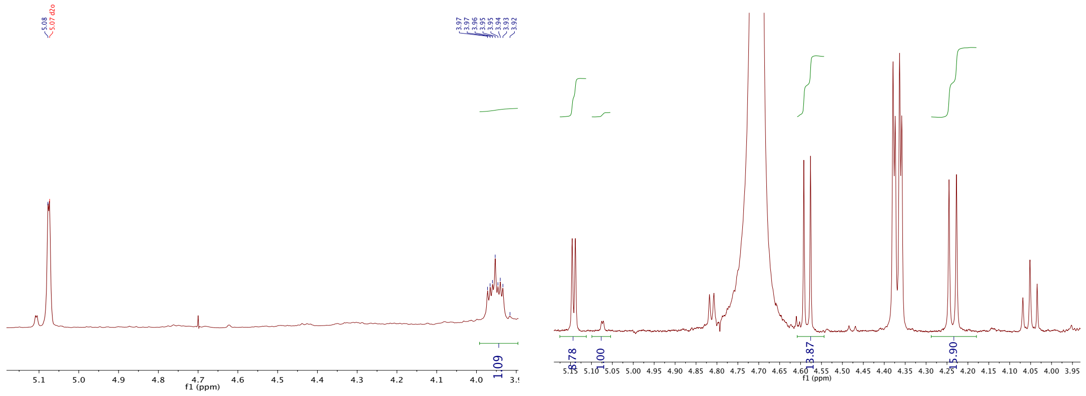

C)

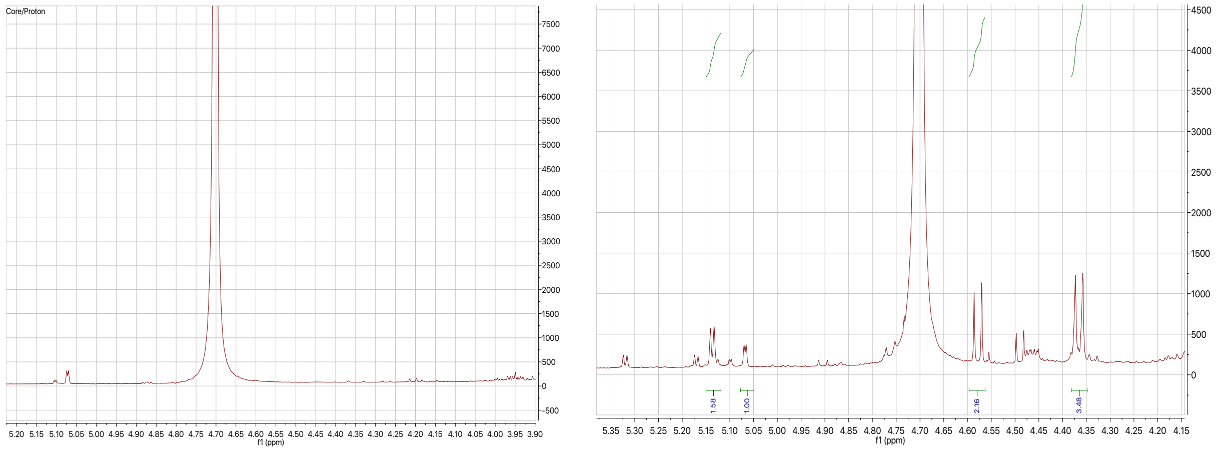

Figure S27: Section of ^{1}H-NMR showing integrals of key signals (lactose anomeric proton δ4.57 ppm/signal from core at δ5.07 ppm) to determine ratios of glycan functionalisation with respect to the different FCD cores. A) FCD **4** (left) and Lactose FCD **6** (right); B) FCD **5** (left) and Lactose FCD **7** (right) and FCD-TTDA derived (left) and Lactose FCD-TTDA[1] (right).

Table S4: Qualitative Ratio of different NMR signal integrals taken from Figure S27. Ratios are calculated by dividing a signal assigned to anomeric proton of lactose with respect of a signal assigned to the core (found on unfucntionalised FCDs **4**, **5** and TTDA-derived FCD[1]).

Glycan-CD species	Ratio
	Glycan / core
Lactose-FCD **6**	20.3±5
Lactose-FCD **7**	13.9±2
Lactose-FCD (TTDA -derived)[1]	2.16±0.5

It is important to take into account the dynamics of the system, the size and 3D presentation of each glycan and the propensity of the conjugated sugars to adopt different conformation in solution. Anomeric signals used for the integration are representative of the glycan population present (only the major anomeric signal for each CD-conjugate were considered as signal overlap of minor anomeric signals associated to a small population of glycan conformers present in solution made their accurate integration impossible).

The data suggests an increase of lactose functionalization for FCDs **4** and **5** when compare to lactose-functionalised FCDs that were prepared using TTDA as the diamine.[1]

FCD Formation NMR monitoring

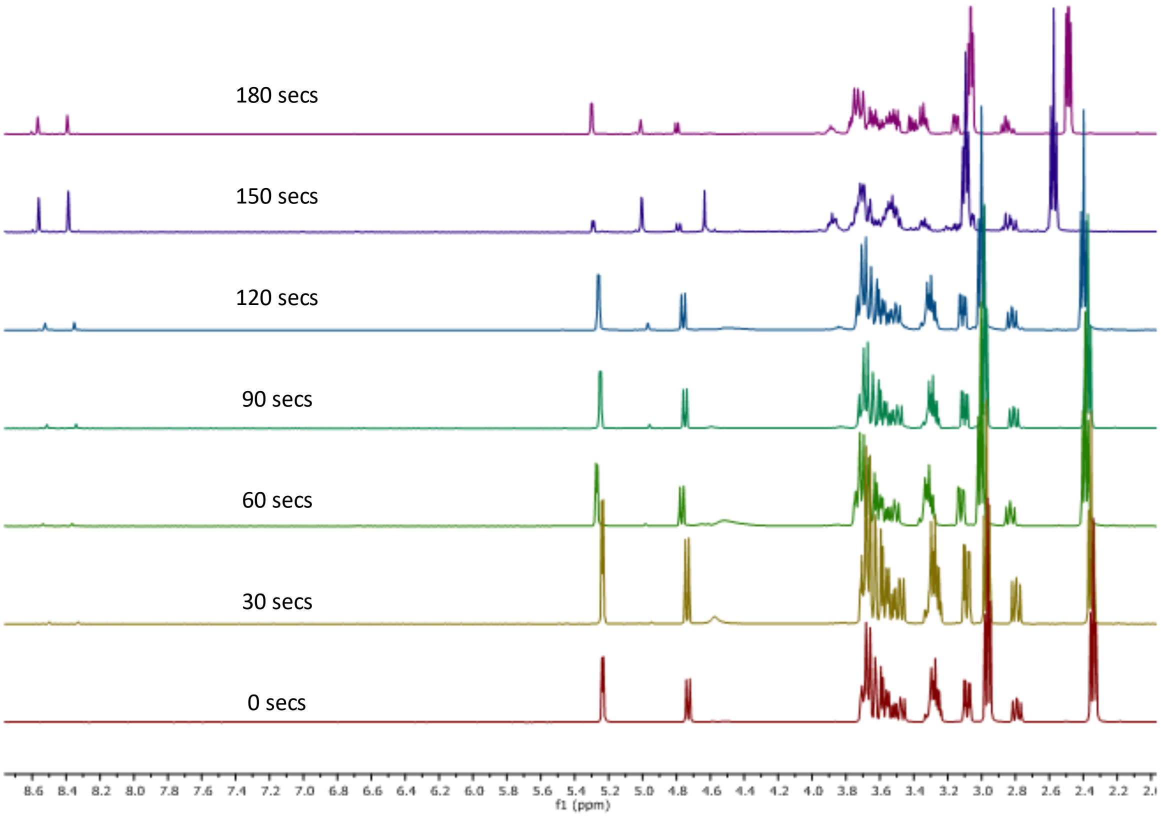

Figure S28: ^{1}H NMR reaction monitoring of COOH-FCD **5** up to 180 secs highlighting significant spectral differences post 120 s (500 MHz, D_2O)

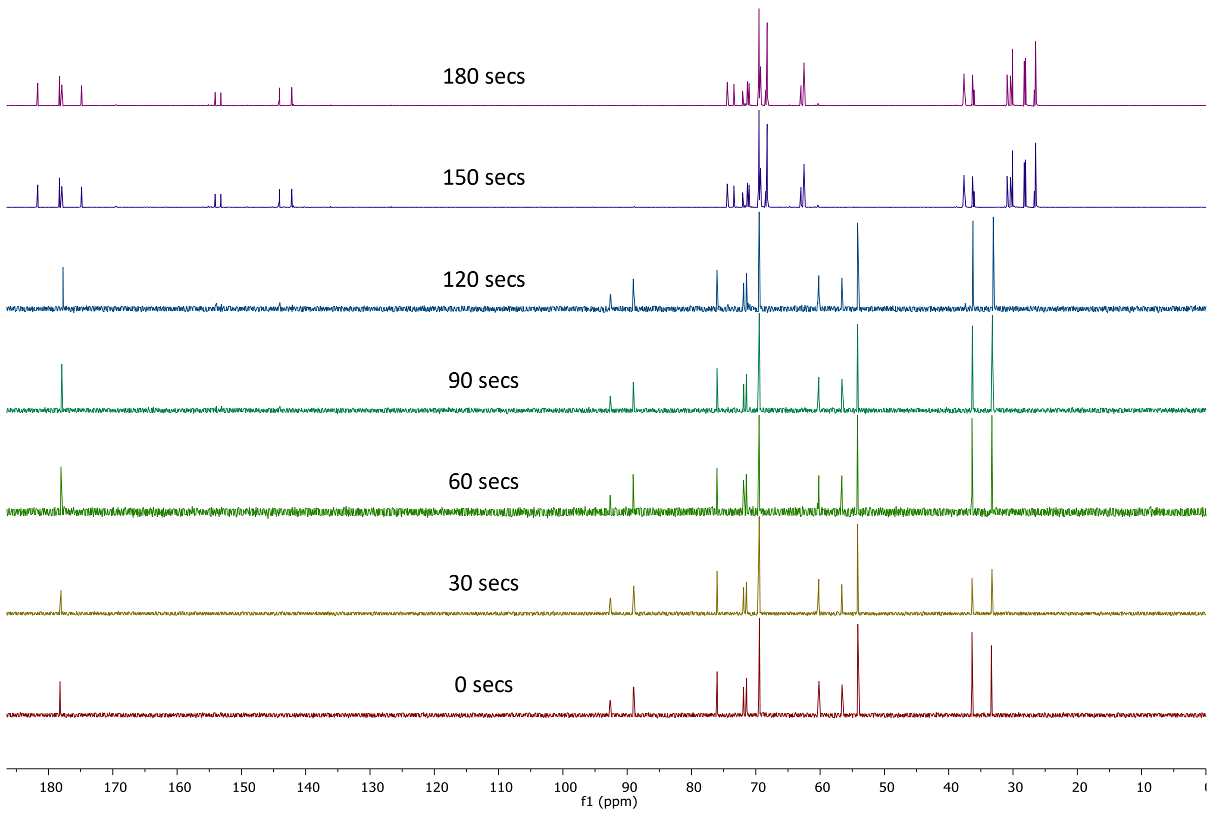

Figure S29: ^{13}C NMR reaction monitoring of COOH-FCD **5** up to 180 s highlighting significant spectral differences post 120 s (125 MHz, D_2O)

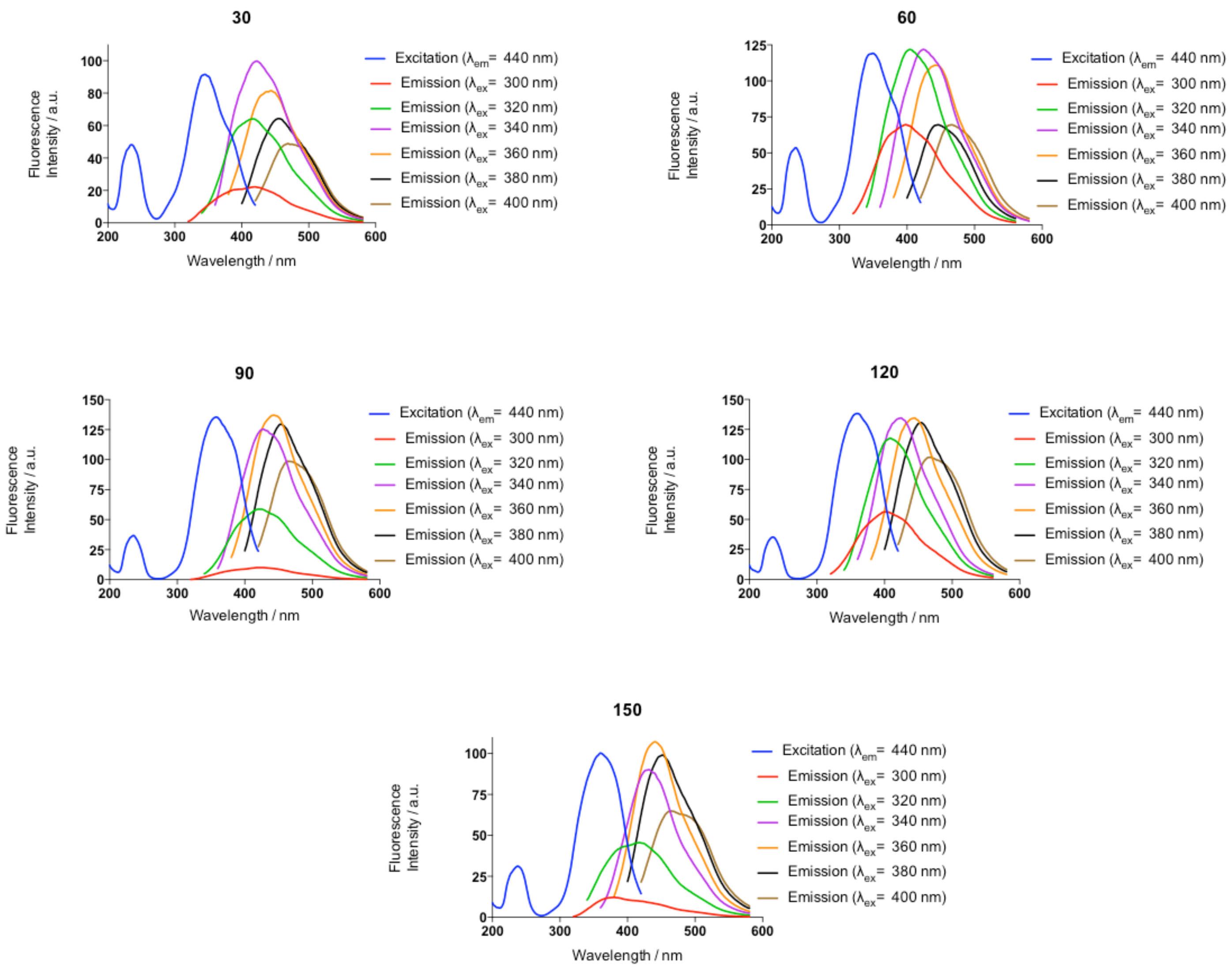

Figure S30: Photoluminescence monitoring of COOH-FCD **5** at different time points from 30 s to 150 s at different excitation wavelengths. No significant changes in the emission profile of **5** was observed at any excitation wavelength, with the exception of ex=300 which was optimum at 120 s. These results suggest that fluorophores contributing to the PL properties of the material are generated early on in the synthesis with an optimum profile at 120 s.

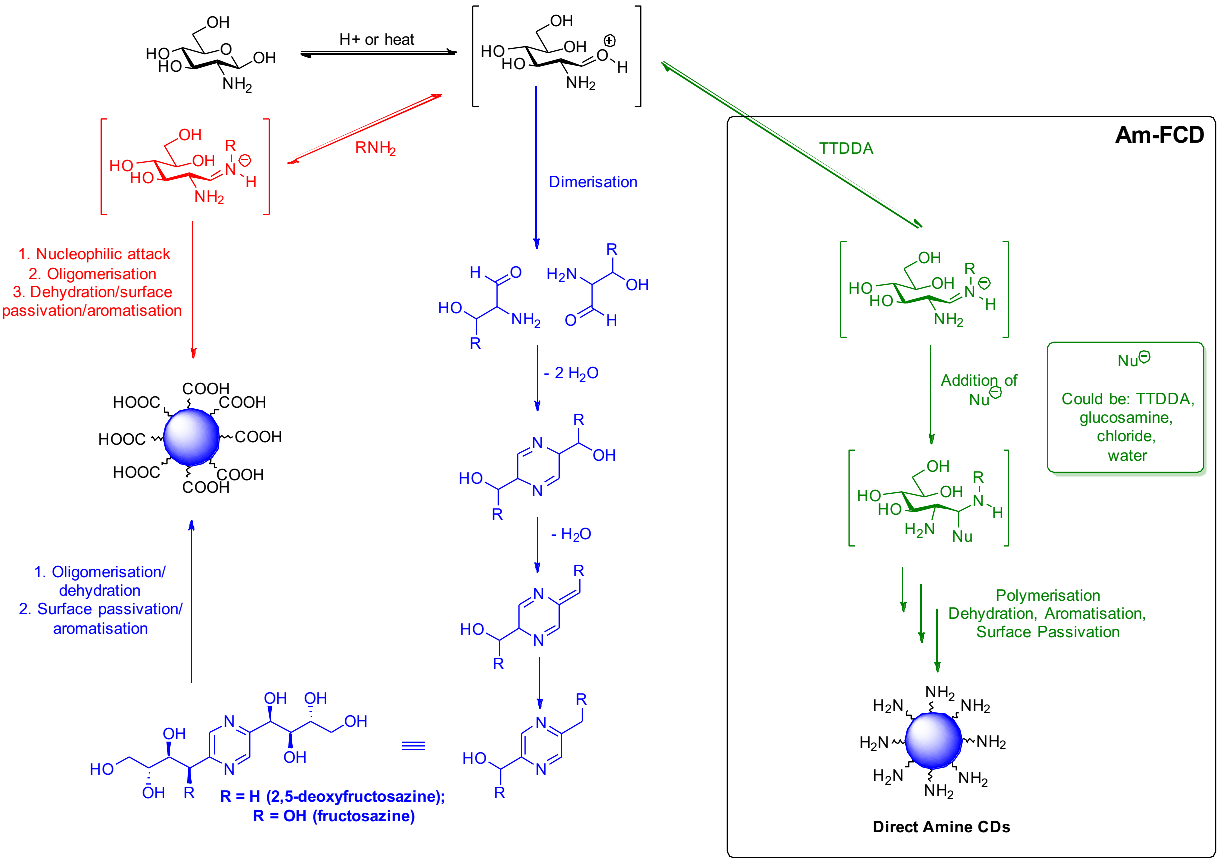

Figure S31: Proposed mechanism of COOH-FCD formation proceeding via head-to-tail dimerization of ring-opened glucosamine via a key pyrazine intermediate (blue) and iminium-mediated oligomer dehydration (red)

COOH-FCD Metal Detection Studies

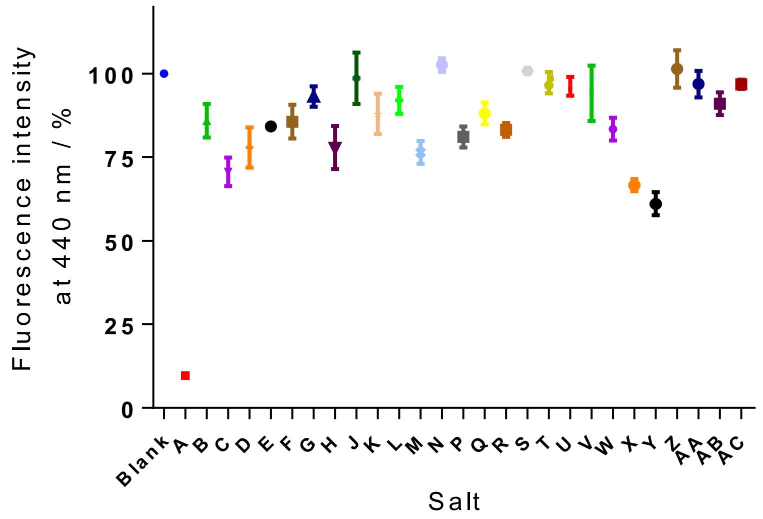

A = $FeCl_3$	B = $FeCl_2$	C = CuCl	D= $CuCl_2$	E = $MnCl_2$	F = $MgCl_2$	G = $CaCl_2$
H = $AgNO_3$	J = CdO	K = Cs_2CO_3	L = CsF	M = CuI	N = CuO	P = KBr
Q = KI	R = KNO_2	S = $LaCl_3$	T = $MgBr_2$	U = Na_2CO_3	V = Na_2HPO_4	W = NaI
X = NaN_3	Y = $NaNO_2$	Z = NaOAc	AA = NH_4Cl	AB = $NiCl_2$	AC = $Ni(OAc)_2$	

Figure S32: Fluorescence emission intensity of **4** (λ_{ex}= 340 nm, λ_{em}= 440 nm) as a function of metal salt concentration (500 µM). Fluorescence is presented relative to 0M

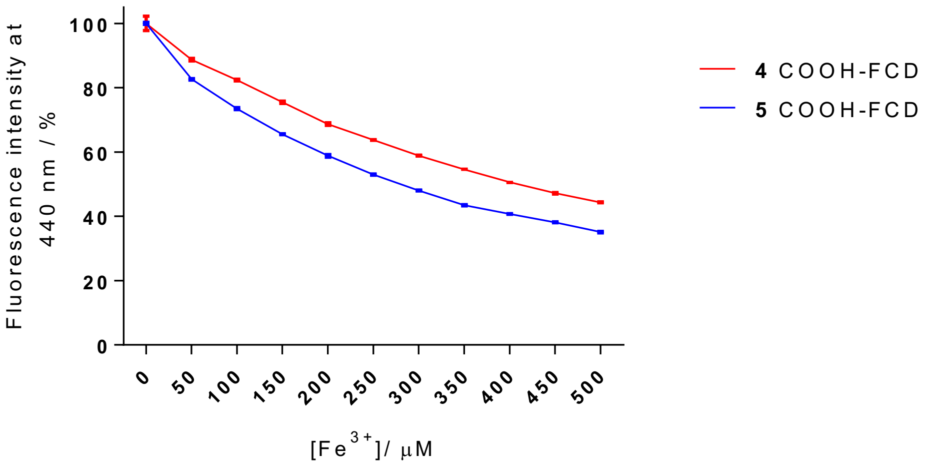

Figure S33: Comparison of **4** and **5** fluorescence quenching (λ_{ex}= 340 nm, λ_{em}= 440 nm) as a function of Fe^{3+} concentration (0-500 µM)

Cell Toxicity Studies

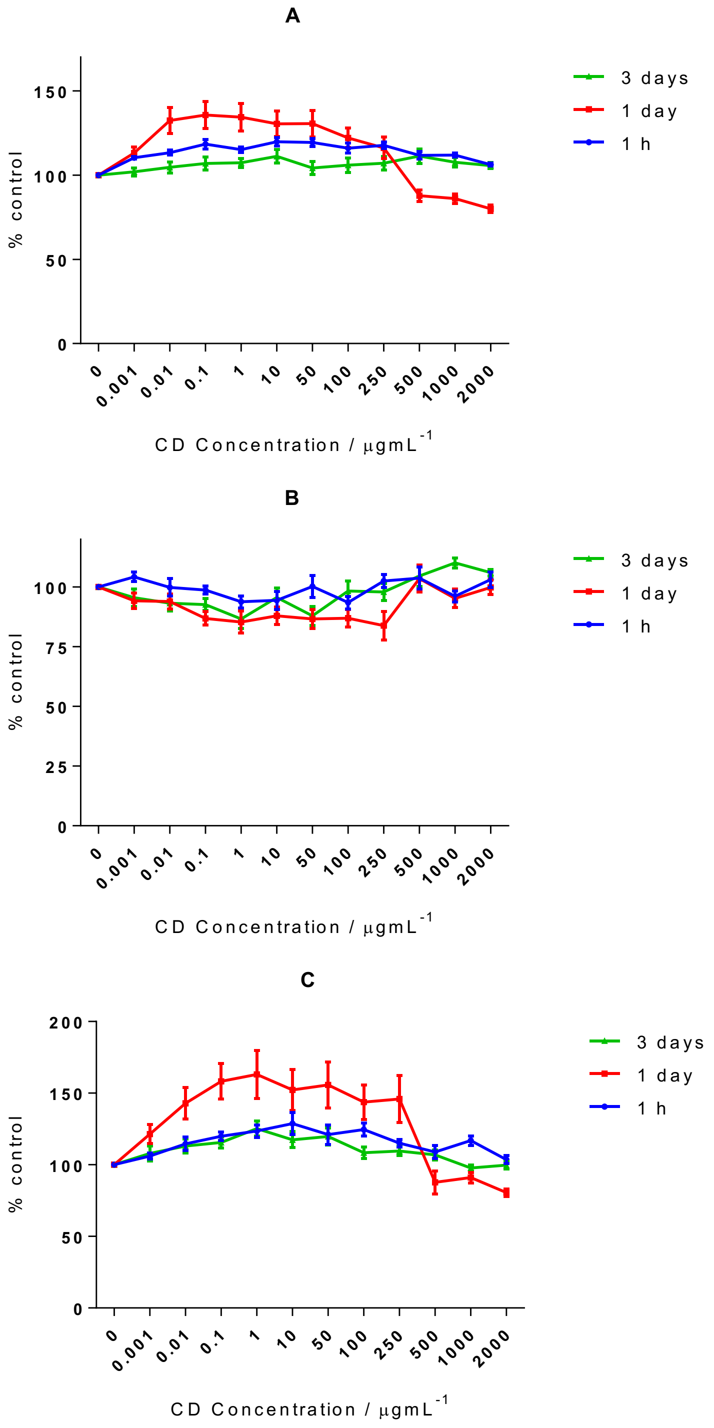

Figure S34: MDA-MB-231 exposure to COOH-FCD **4** – (A) reductive metabolism; (B) viable cell numbers and (C) reductive metabolism (RM) per cell compared to untreated cells

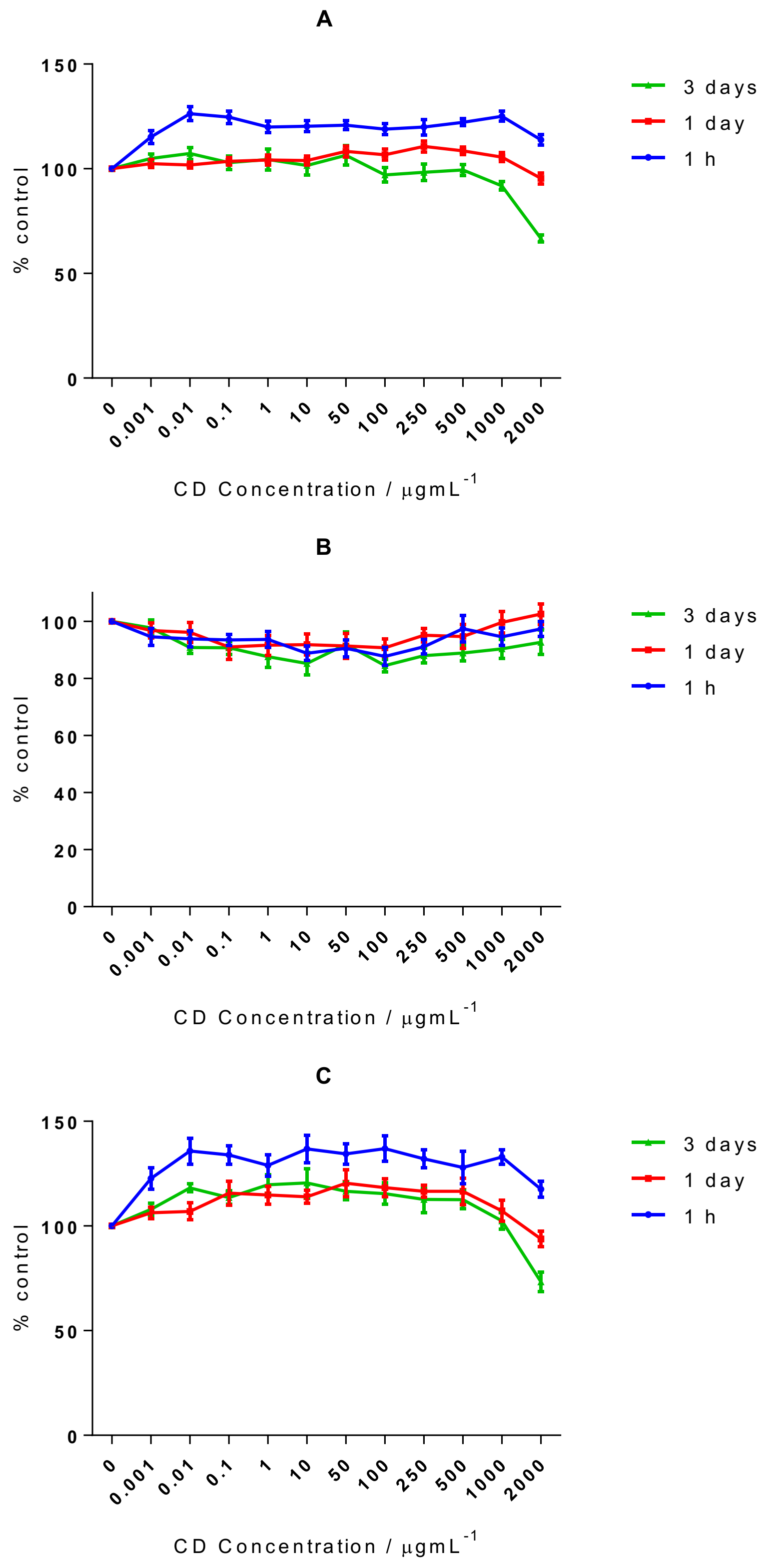

Figure S35: MDA-MB-231 exposure to COOH-FCD **5** – (A) reductive metabolism; (B) viable cell numbers and (C) reductive metabolism (RM) per cell compared to untreated cells

Cell Internalisation and Confocal Microscopy Images

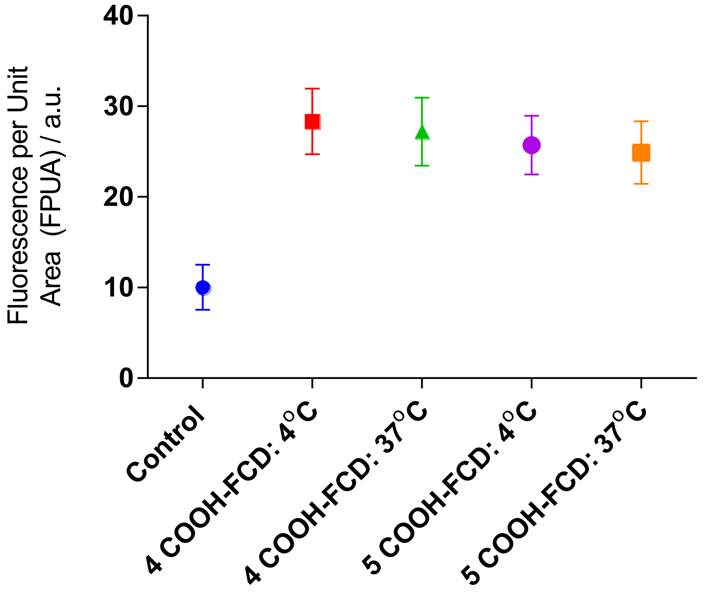

Figure S36: Fluorescence per unit area of differentially functionalised COOH-FCD inside MDA after exposure at 500 µgmL^{-1} for 2hrs at 4°C and 37°C

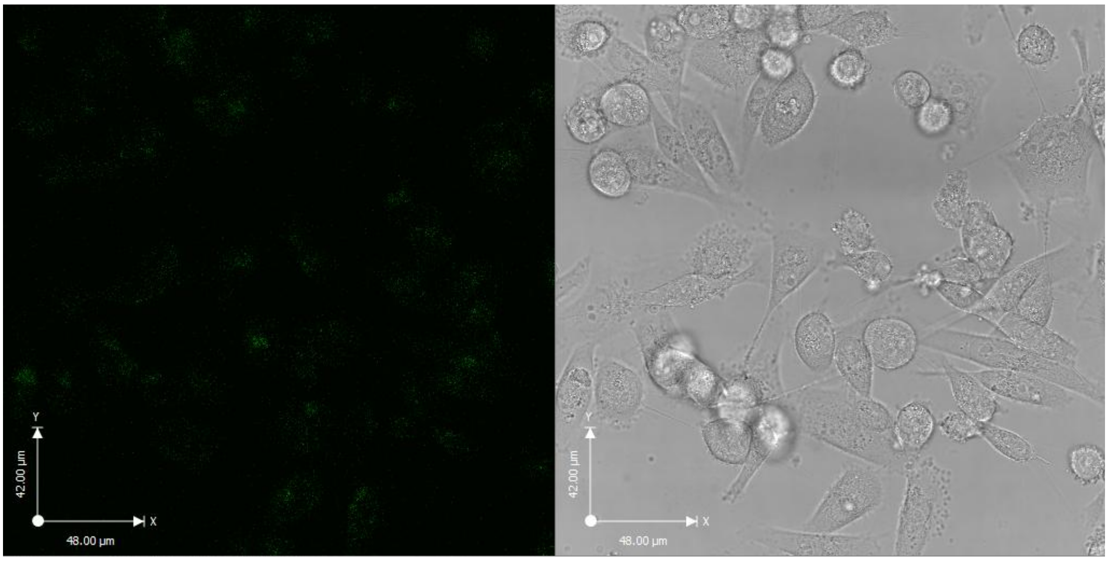

Figure S37: Confocal images of untreated MDA-MB-231 cells

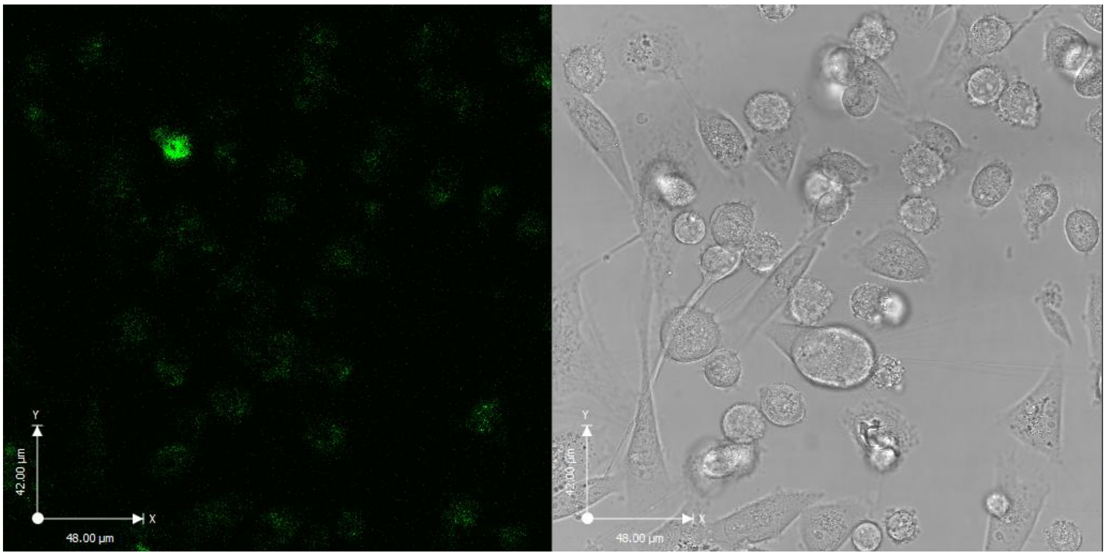

Figure S38: Confocal images of MDA cells exposed to 500 µgmL^{-1} of **5** for 2h at 37°C

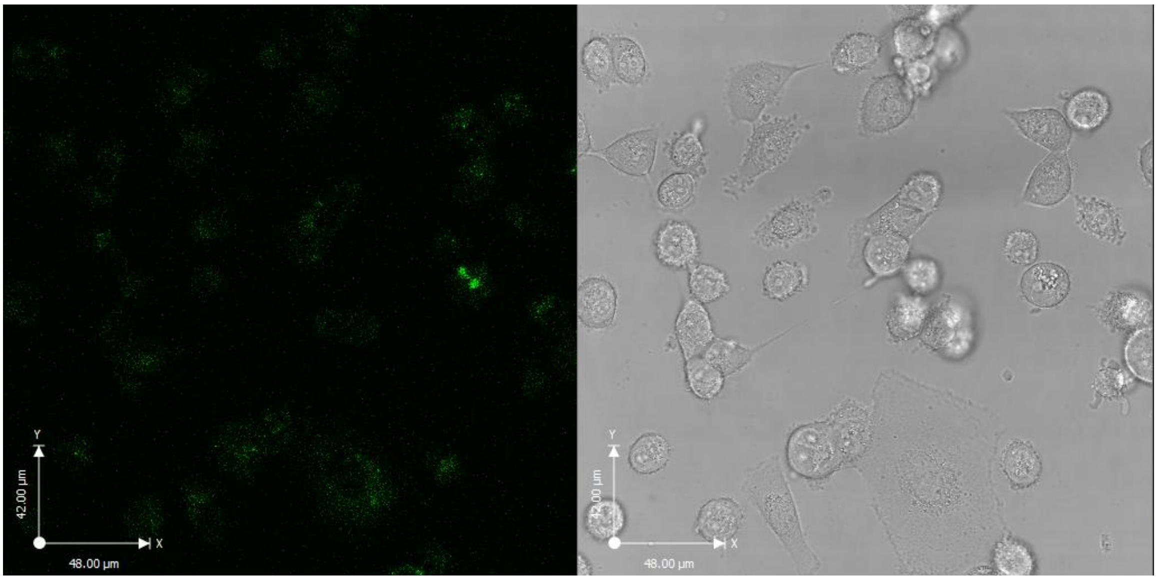

Figure S39: Confocal images of MDA cells exposed to 500 µgmL^{-1} of **5** for 2h at 4°C

References:

1. Hill, S. A.; Benito-Alifonso, D.; Morgan, D. J.; Davis, S. A.; Berry, M.; Galan, M. C., Three-minute synthesis of sp3 nanocrystalline carbon dots as non-toxic fluorescent platforms for intracellular delivery. *Nanoscale* **2016,** *8* (44), 18630-18634.
